# Human Activity Recognition: A Dynamic Inductive Bias Selection Perspective

**DOI:** 10.3390/s21217278

**Published:** 2021-11-01

**Authors:** Massinissa Hamidi, Aomar Osmani

**Affiliations:** LIPN-UMR CNRS 7030, Université Sorbonne Paris Nord, 93430 Villetaneuse, France

**Keywords:** human activity recognition, inductive bias, meta-learning, hyperparameter optimization, sensor-rich environments, sensor characteristics, sensor deployments, body sensor networks

## Abstract

In this article, we study activity recognition in the context of sensor-rich environments. In these environments, many different constraints arise at various levels during the data generation process, such as the intrinsic characteristics of the sensing devices, their energy and computational constraints, and their collective (collaborative) dimension. These constraints have a fundamental impact on the final activity recognition models as the quality of the data, its availability, and its reliability, among other things, are not ensured during model deployment in real-world configurations. Current approaches for activity recognition rely on the activity recognition chain which defines several steps that the sensed data undergo: This is an inductive process that involves exploring a hypothesis space to find a theory able to explain the observations. For activity recognition to be effective and robust, this inductive process must consider the constraints at all levels and model them explicitly. Whether it is a bias related to sensor measurement, transmission protocol, sensor deployment topology, heterogeneity, dynamicity, or stochastic effects, it is essential to understand their substantial impact on the quality of the data and ultimately on activity recognition models. This study highlights the need to exhibit the different types of biases arising in real situations so that machine learning models, e.g., can adapt to the dynamicity of these environments, resist sensor failures, and follow the evolution of the sensors’ topology. We propose a metamodeling approach in which these biases are specified as hyperparameters that can control the structure of the activity recognition models. Via these hyperparameters, it becomes easier to optimize the inductive processes, reason about them, and incorporate additional knowledge. It also provides a principled strategy to adapt the models to the evolutions of the environment. We illustrate our approach on the SHL dataset, which features motion sensor data for a set of human activities collected in real conditions. The obtained results make a case for the proposed metamodeling approach; noticeably, the robustness gains achieved when the deployed models are confronted with the evolution of the initial sensing configurations. The trade-offs exhibited and the broader implications of the proposed approach are discussed with alternative techniques to encode and incorporate knowledge into activity recognition models.

## 1. Introduction

Activity recognition aims to provide accurate and opportune information based on people’s activities and behaviors [1]. It is of utmost importance in many applications ranging from patient monitoring systems [2], ambient assisted living [3], etc. Tracking daily activities and providing, for example, real-time feedback to patients with obesity, diabetes, or cardiovascular diseases as well as up-to-date reports to clinicians has the potential to enhance the health system [4,5,6,7]. Similarly, energy consumption in large infrastructures and housings could be monitored and regulated based on the real-time tracking relative to human activities [8].

Sensor rich environments. A growing number of domains is witnessing the development of sensor-rich environments powered by the ever-increasing pervasiveness of sensing devices. An important notion that characterizes these environments is that of *coverage* provided by the sensing nodes encompassing the deployment. This notion is essential for allowing the capture of user movements in an unambiguous manner. In this sense, integration of the diverse sensing modalities plays a key role via three broad goals: increasing completeness, conciseness, and correctness of data [9]. This notion of coverage is linked to (i) the intrinsic capabilities of each sensor to cover a surface and the way this is performed. It is then linked to (ii) the way different sensing nodes are placed on the body (in the case of body deployments) and in the environment in which the user has to evolve (in the case of non-corporeal deployments). This poses roughly two challenges: the first one is related to the sensors’ placement (and displacement from an initial configuration) [10], and the second is related to the heterogeneity of the sensors deployments [11], i.e., the proprietary and non-proprietary solutions (both in on-body and non-corporeal deployments) that hinder the process of integration. Both issues have an important impact on the notion of coverage which determines the quality and robustness of the final activity recognition models.

Constraints related to the sensing devices. In addition to coverage issues, many kinds of constraints related to sensing devices have to be taken into account when designing activity recognition models. These constraints fall generally into three major parts, which can be organized in a bottom-up fashion. The first includes sensing constraints related to the intrinsic characteristics of the sensing devices (precision, sensitivity, dynamic range, thermal drift, etc.) [12]. The second includes energy and computational constraints that are related to the individual sensing nodes and the way they modulate the measurement (sampling frequency, wake-up/sleep modes, etc.) in order to comply with these constraints [13]. The third includes the collective dimension of the sensor deployments where many different challenges related to transmissions arise including power, computation, security and interference, material constraints, robustness, continuous operation, and regulatory requirements [14,15]. Along with the coverage issues, these constraints have an important impact on the final activity recognition models as the quality of the data, its availability, and its reliability, among other things, are not ensured.

Dynamic selection of inductive biases. Current approaches for activity recognition are based on the activity recognition chain [16], which defines several steps that the sensed signals undergo. This is an inductive process that involves exploring a hypothesis space to find a hypothesis (or theory) able to explain the observations. Often, the hardest problem in this process is how to choose the hypothesis space in such a manner that it contains a satisfactory hypothesis towards which we can converge rapidly, i.e., using a small number of examples [17]. The choice of inductive biases, e.g., preprocessing filter, segment size, feature set, etc., has a significant impact on this problem. In addition to the challenges of coverage and the constraints related to detection devices, environments rich in sensors and the phenomena to be detected are subject to change during the actual deployment of activity recognition models in real situations. While setting inductive biases that apply to specific problems may be of benefit in controlled environments, performing this operation during the early stages of the activity recognition chain in real-world environments will inevitably result in inefficient exploration of the hypotheses space. Worse yet, the final hypothesis that will be chosen may not explain the learning examples. A natural solution is to delay the selection of the inductive biases as late as possible and to maintain competing hypotheses able to quickly adapt to new situations by means of a dynamic selection of the inductive biases. This results in different implications from an operational point of view, namely, maintaining a set of inductive bias alternative candidates (the domain) and rapidly exploring the space in order to elect the appropriate hypothesis (amount of supervision with learning examples). In other words, in order to be efficient, the exploration of the hypothesis space must be structured by exploiting a priori knowledge on the deployments of sensors and the phenomenon itself.

Use-case and evaluation. In order to illustrate the advantages of the dynamic selection of inductive biases, we present a use-case pertaining to the SHL dataset [18], one of the most recent and featured datasets in human activity recognition literature. This dataset is a highly versatile and is a precisely annotated dataset dedicated to mobility-related human activity recognition (3000 h of locomotion data). In contrast to related representative datasets such as [19,20,21,22], the SHL dataset provides a sensor-rich environment featuring, simultaneously, multimodal and multilocation locomotion data recorded in real-life settings. We evaluate a first model based on the traditional activity recognition chain instantiated by using neural networks-based architectures. We then illustrate the dynamic inductive bias selection using the proposed approach based on the optimization of architecture’s hyperparameters [23]. Extensive experiments make the case for the proposed meta-modeling approach and show the robustness gains achieved when the deployed models are confronted with evolution of the initial sensing configurations (ablation of an increasing number of sensors from the initial deployment). In particular, the incorporation of derived knowledge about the sensors’ deployment allows easy adaptation using little or no supervision at all.

Organization of the paper. This paper builds upon and extends our previous work on human activity recognition [23,24,25] and is organized as follows. In Section 2, we introduce the context of human activity recognition, the activity recognition chain, and we clarify the scope of our work. Section 3 focuses on the notion of coverage characterizing the sensor deployments and their topologies. In Section 4, we describe the proposed metamodeling approach. Section 5 describes the case study and the used dataset while the evaluation results are presented in Section 6. Detailed discussions of the proposed approach and future directions are provided in Section 7, and Section 8 concludes this manuscript.

## 2. Human Activity Recognition

There are various types of human activities. Depending on their complexity, the authors in [26] categorized human activities into four different levels: *gestures*, *actions*, *interactions*, and *group* activities. In this paper, we focus on the two first levels. Gestures, e.g., stretching an arm and raising a leg, are elementary movements of a user’s body part, while actions, e.g., walking and waving, are a sequence of multiple gestures organized temporally. Interactions and group activities involve multiple users and objects and can be tackled via the composition of models describing the former two levels.

Many different approaches have been introduced in the literature to tackle human activity recognition. These approaches differ in terms of the type of sensing strategies that are used to capture body movements. These approaches can be categorized into (i) radio-frequency/device-free, (ii) vision and depth images, and (iii) inertial sensors. Device-free activity recognition refers to the use of the signals generated by standard wireless equipment to capture users’ movements in a non-invasive manner [27]. Vision and depth images-based methods utilize spatio-temporal characteristics extracted from video sequences and the 3D motion feature to describe the action [28]. In the case of inertial sensor-based approaches, on-body sensors placed in different parts of the body generate streams of observations, such as acceleration, which describe similarly the performed actions. In this paper, we are mainly interested in the latter approach, but the proposed perspective can apply similarly for the two other ones.

**Figure 1:**
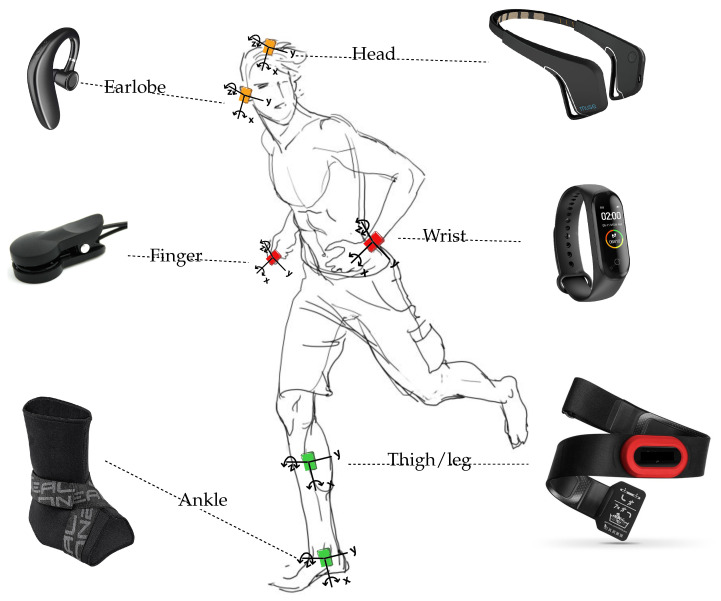
Examples of concrete wearable devices, along with their typical on-body location, can be found in body area networks dedicated to patient monitoring. Both vital signs and body movements can be captured by these kinds of devices.

For example, Figure 1 illustrates a body area network dedicated to patient monitoring and encompassing various sensing nodes responsible for capturing the vital signs as well as the patient’s activities. This figure also illustrates a set of wearables for biomedical sensing, including activity trackers, smartwatches, smart clothing, patches/tattoos, and ingestibles/smart implants. In addition to medical applications, many different applications observe the opportunity of leveraging the context provided by human activity recognition models including assisted living and home monitoring, and sports and leisure applications. We refer the reader to the literature review performed in [29].

### 2.1. Background on Activity Recognition Chain

**Figure 2:**
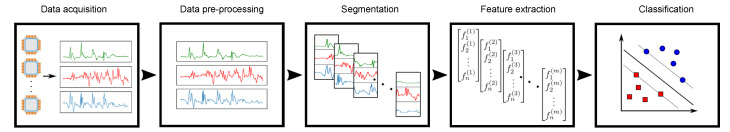
Activity recognition chain defined in [16] which includes the following (from **left** to **right**): data acquisition, signal preprocessing, segmentation, feature extraction, classification, and evaluation stages. From [30].

The activity recognition chain [16] is a widely used machine learning-based inductive process in the literature that is used to model human activities (our phenomenon of interest). It is composed of five different steps: *data acquisition*, *preprocessing*, *segmentation*, *feature extraction*, and *classification*. Figure 2 illustrates the steps of the activity recognition chain as defined in [16]. As presented in the following, the goal of these steps is to build a model capable of recognizing human activities (outputs) from the streams of observations (inputs).

#### 2.1.1. Data Acquisition

**Figure 3:**
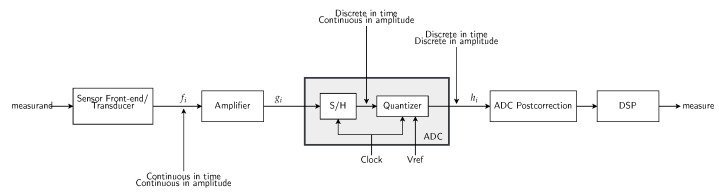
Schematic of the typical units that compose a sensor (from **left** to **right**): transducer, amplifier, analog-to-digital converter (ADC), ADC postcorrection, and digital signal processing (DSP) units. The measurement of a phenomenon (measurand) as simple as temperature by a sensor is in and of itself an inductive process involving many biases. The action of the physico-electrical process of the sensor (via the units it encompasses) generates an electrical signal proportional to the physical phenomenon being measured. We, actually, do not have access to the physical phenomenon itself but to a representation provided through a transfer function deduced mathematically and that is specific to the physico-electrical process of the sensor. The choice of this process constitutes a bias similar to the elaboration of the transfer function.

**Figure 4:**
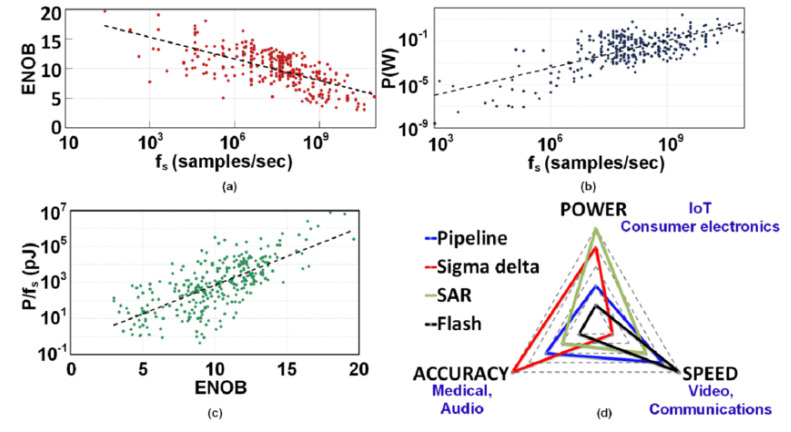
The analog-to-digital converter is one of the units encompassing the sensors. Its design accounts for various trade-offs which ultimately impact generated measurements and performances. Trade-offs in conventional analog-to-digital converter architectures between (**a**) speed and accuracy, (**b**) speed and power, and (**c**) accuracy and energy, as reported in [51]. (**d**) Spider diagram of analog-to-digital architectures (different color lines), design trade-off, and associated applications (in blue). From [52].

Given a collection $S=\{s_{1},\ldots ,s_{M}\}$ of *M* sensors (also referred to as data generators, data sources, or data acquisition systems) carried by the user during daily activities to capture the body movements, each sensor $s_{i}$ generates a stream $x^{i}=\left(x_{1}^{i},x_{2}^{i},\ldots \right)$ of observations of a certain modality, which can comprise several channels, e.g., the accelerometer modality contains three channels corresponding to *x*, *y*, and *z* axes. The data acquisition step encompasses many aspects, including the following: (1) The intrinsic characteristics of the sensors, which are determined by their various components involved in transforming the sensed phenomenon into an electrical signal (See Figure 3). In particular, as depicted in Figure 4, the design of analog-to-digital (ADC) converters obeys a trade-off involving simultaneously conversion accuracy, transformation speed, and power, which ultimately results in mitigating some hard-coded inductive biases in the activity recognition chain.

The spatial structure of the sensors deployment and the induced views and the phenomena being monitored (human activities in our case) are accentuated by the sensors’ capabilities and the perspectives (views) through which the data are collected (position in space, position on the body, video capture modalities, acceleration, gravity, etc.) [23,31,32,33]. Moreover, the incomplete and redundant perspectives can confuse the concepts between them and reduce the performance of the learning independently of the algorithm used. This aspect is further detailed in Section 3; (3) Often, in addition to being involved in sensing the phenomenon, the data acquisition systems, in their extended definition as computing platforms, also take part in the subsequent computations of the activity recognition chain. This brings in questions related to the orchestration and optimal deployment of these computations [34].

#### 2.1.2. Preprocessing

In this step, the streams of observations generated by each sensor are “enhanced” in the perspective of the features extraction step that follows in the activity recognition chain. The preprocessing step renders the features extraction step more robust by, e.g., removing outliers, boosting frequencies or portions of the spectrogram, canceling noise components of the signal, etc. In the case of device-free activity recognition approaches, the authors in [35,36], for example, provide a comprehensive list of preprocessing methods widely used in the literature, each of which can be suitable in different situations and involves trade-offs. Some of these methods include High-pass filter (pre-emphasizing), Hampel filter [37], Phase sanitization [38], phase calibration [39], Butterworth low-pass filter [40], STFT (Heisenberg uncertainty principle [41]), Savitzky–Golay filter [42], and Birge–Massart filter [43].

#### 2.1.3. Segmentation

During this step, the preprocessed observations streams are divided into segments that will likely contain the activities in its entirety or in part depending on the segmentation procedure and its hyperparameters.

**Figure 5:**
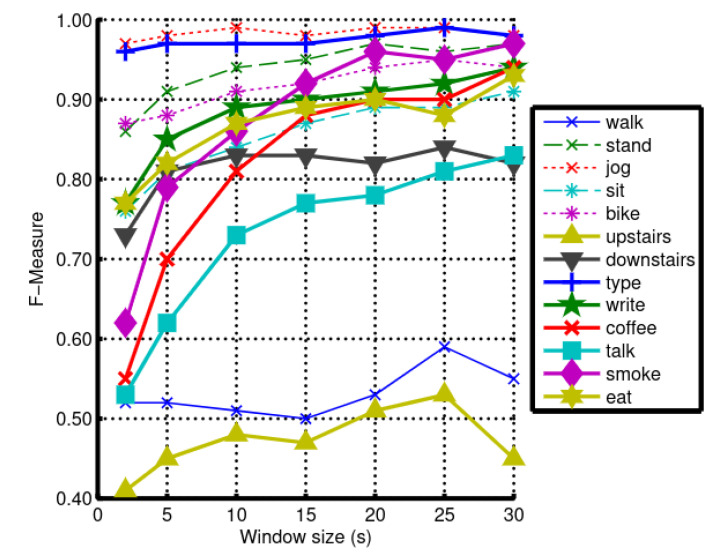
Effect of window size (in seconds) on the recognition performances (F-measure) of an activity recognition model trained on various daily activities such as walk, stand, and jog. Depending on the activity being monitored, the effect of the window size on the final model’s performances varies to a large extent. From [45].

Many different types of segmentation procedures exist in the literature around activity recognition and beyond, including time-based, event-based, and energy-based [44]. Various works studied the effects of different segment lengths on the recognition performances empirically [45,46]. For example, Figure 5 shows the effect of window size on the performances (f-measure) of activity recognition models.

Issues with time-based segmentation are not circumscribed to the choice of the segment’s length but are also tightly linked to the feature extraction step. Activities that last for variable time constitute a critical issue. For example, fixing the segment path can result in spectral leakage that impacts the subsequent steps, which noticeably includes the feature extraction step from the spectral representation of the signal. Indeed, spectral leakage causes the spectrum to be noisy, impacting the correct determination of frequencies, etc. Issues go beyond the impact of the segment’s length on the extracted features. Many studies showed the impact related to the overlap of windows on the classification and evaluation steps [47,48]. A growing line of research considers the issues that stem from the dynamic nature of the sensor deployments regarding segmentation.

#### 2.1.4. Feature Extraction

Features are extracted from the preprocessed segments obtained in the previous steps and not from the entire stream of observations. The resulting features are largely impacted by the hyperparameters controlling the preceding steps. In [49], for example, the authors investigated the influence of preprocessing operations on features extracted from accelerometers in both time and frequency domains. The obtained results indicate that the preprocessing methods have to be carefully chosen as their impact is significant and disparate. Another example is related to the impact of segmentation on the resulting frequency domain representation, which is obtained using the short-time Fourier transform. Indeed, two effects at least can be mentioned: on the one hand, the trade-off between resolution and the Heisenberg uncertainty principle and, on the other hand, spectral leakage [50].

#### 2.1.5. Classification and Evaluation

The final step of the activity recognition chain consists of the classification of each individual segment of features, obtained before, into its correct class. With regards to the inductive process evoked in the introduction, this step corresponds to electing a hypothesis that best explains the learning examples (or the segments of features) that are supplied along with the hypotheses space, i.e., the set of inductive biases ranging from the sensing and deployment models to the learning algorithm that we chose including the preprocessing, segmentation, and feature extraction steps (See Section 4.2 for further details). As stated, setting the hypothesis space in advance and electing a unique hypothesis, which is supposed to hold during the entire model deployment in real-world environments, are not suitable procedures. For example, the authors in [53] were interested in the highly dynamic nature of wearable sensor deployments in the case of health monitoring, where changes in the data acquisition step, i.e., sensing platform (e.g., sensor upgrade) and platform settings (e.g., sampling frequency and on-body sensor location), cause activity recognition models to degrade in terms of performances.

In order to confront these aspects, we take a metamodeling approach where the aforementioned inductive biases are exhibited and handled by using suitable methods, which are detailed in the following sections. We focus particularly on metamodeling the data acquisition part of the activity recognition pipeline. The following section will describe aspects related to the sensors deployments: their topology, structure of interactions, sensor coverage, heterogeneity, etc.

## 3. Sensor-Rich Environments

The coverage problem in wireless sensor networks can be broadly defined as a measure of how efficiently a phenomenon is monitored by the sensor nodes. This issue has generated much interest over the years and resulted in the definition of many coverage protocols [54]. The notion of coverage is linked to (i) the intrinsic capacities of each sensor to cover a surface and how this is conducted. It is also linked to (ii) the way in which the various sensors are placed on the body, in the case of on-body deployments, or with respect to the environment in which the user is supposed to operate, in the case of non-corporeal deployments. We will explore these aspects by using deployment examples.

This section illustrates some examples of sensor-rich environments, noticeably on-body sensor deployments used in the context of human activity recognition. We explore an essential notion that defines the coverage score of a given sensor deployment, namely the sensing capabilities of individual sensory nodes. We also explore the collective dimension of the sensors materialized by the topology or the placement of the sensing nodes in space. We will focus on the placement of sensors in the case of on-body deployments and the long line of research that has studied this aspect.

### 3.1. Examples of on-Body Sensor Deployments

**Figure 6:**
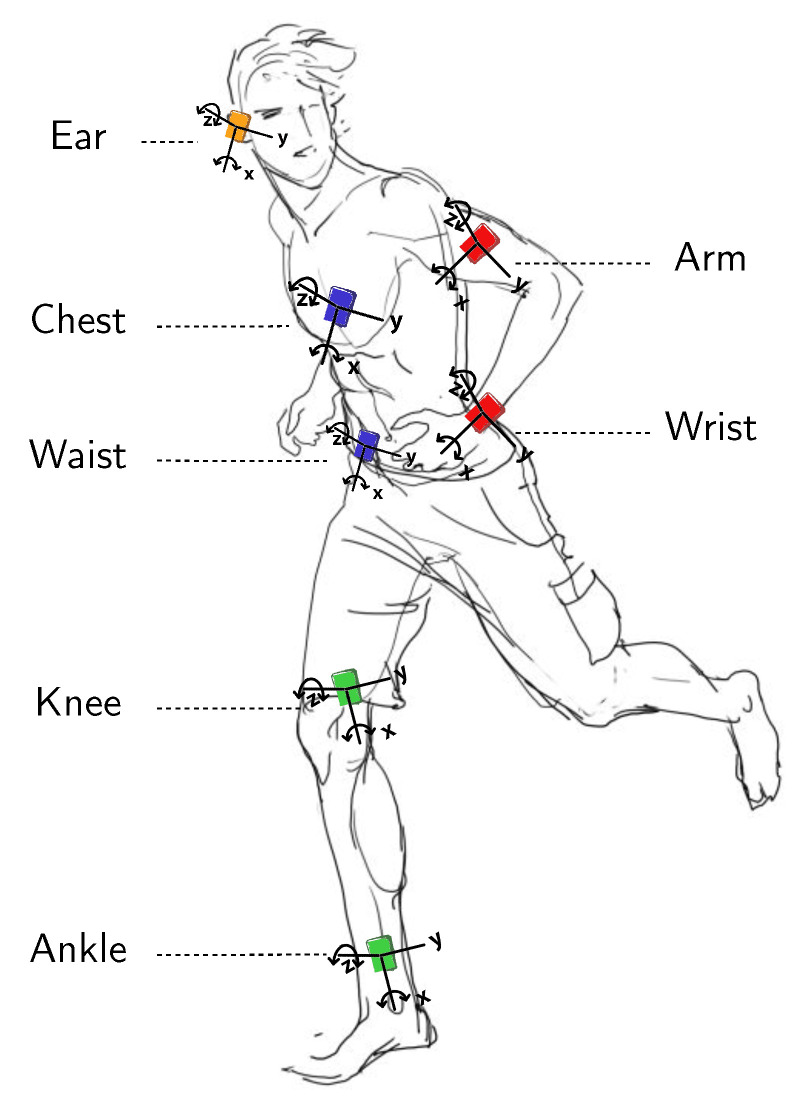
Various locations of wearable devices on the body. Illustration of a subject wearing the wearable sensors on the ear, chest, arm, wrist, waist, knee, and ankle.

Within the framework of the recognition of human activities, sensors are generally placed on the following body positions: *waist*, *thigh*, *necklace*, *wrist*, *chest*, *hip*, *lower back*, *trunk*, *shanks*, *ankle*, *pocket*, *hand*, *back pack*, *torso*, *ear*, etc. (see Figure 6). A long line of research work has focused on the problem of optimal placement and combination of sensors on the body in order to achieve satisfactory levels of recognition, and many reviews report on this, such as [10,55]. As an example, Gjoreski et al. [56] studied the optimal location of accelerometers among waist, chest, thigh, and ankle for posture recognition and fall detection. The authors found that several sensor configurations are sufficient to recognize most postures and fall events correctly. More generally, several works, e.g., [10,57,58,59,60,61,62], provided empirical evidence on the substantial improvements obtained using accelerometer placed on the waist for the recognition of many activities such as sitting, standing, walking, lying in various positions, running, stair ascent and descent, vacuuming, and scrubbing. Additionally, acceleration data generated by this particular position are identified to be invariant across positions [63].

In many empirical evaluations comparing multi-sensor versus single-sensor deployments for activity recognition, e.g., [64,65], the settings leveraging multiple sensors tend to perform far better than their counterparts. However, different on-body locations and their various combinations for activity recognition result in varying performances, and no consensus tends to emerge.

### 3.2. Sensor Deployment Topology

Sensor deployment topology (or the collective dimension of the sensors) is essential for activity recognition models. It defines the coverage model for optimal data acquisition. It ensures redundancy, robustness, and data security. It is also crucial in wireless sensor networks by its impact on node energy, communication bandwidth, and quality of service [66].

#### Sensing Capabilities (Space Coverage)

Each sensor node has a limited sensing range and can only cover a limited physical area of the network field. Sensing models are abstraction models that are used to reflect the ability of the sensors to perceive the phenomenon of interest as well as the quality of the generated measures. Indeed, depending on the sensing model featured by a given sensor, the activity recognition model would have access to partial aspects of the monitored phenomenon. For example, the sensing models can be classified into either directional or omnidirectional sensing models based on the direction of the sensing range. Moreover, based on the sensing ability, sensing models can be broadly classified into deterministic and probabilistic sensing models [54].

#### Reconciling Various Views/Perspectives

The placement of sensors makes it possible to provide various perspectives, and the use of several modalities makes it possible to provide several points of view. Here, the problem is to define the appropriate locations for each modality [23]. The problem is more complex than in the case of non-corporeal deployments because the positions of the sensors between them change according to the movements. This can generate ambiguity and misinterpretations if the relative movements of the sensors between them are not taken into account.

#### Sensors Placement and Displacement

Even if the wearable sensors should be correctly attached to the body, vibration or displacement (both intentional and unintentional) of those sensors cause signal interference and, thus, the deterioration of measurement accuracy [10]. Various studies were conducted in the literature and different approaches were proposed to cope with these issues [10,67,68,69,70,71]. For example, the authors in [68] considered the robustness of activity recognition models relative to sensor displacement and proposed a set of heuristics that allows the implementation of displacement-tolerant activity recognition models. Similarly, in [69], the authors investigated how various sensor displacement scenarios (ideal-placement, self-placement by the user, and induced displacement) impact the performances of activity recognition models.

#### Heterogeneity of Deployments

Another problem is related to the lack of interoperability among different sensor deployments. This problem is, in particular, due to the existence of different incompatible solutions (owners and non-owners). This makes it difficult both to integrate new deployments and their constant evolution [11,72,73]. Another source of heterogeneity is related to the incompatibility of detection solutions. In [72], the authors investigated in a systematic manner sensor-specific, device-specific and workload-specific heterogeneities using 36 smartphones and smartwatches consisting of 13 different device models from four manufacturers. Their results indicate that on-device sensor and sensor handling heterogeneities significantly impair the performances of activity recognition models.

### 3.3. Variety of Sensing Modalities

In addition to the on-body sensor placement, which we saw above substantially impacting the performances of activity recognition models, the sensing modalities, such as acceleration, gravity, ambient pressure, etc., are also impactful and, thus, of utmost importance for the design of sensor-rich environments. In a similar manner with the on-body sensor placement, sensing modalities are found to be beneficial when sensor-rich environments simultaneously provide them in multitudes. One of the predominant sensing modalities used in the literature is obviously acceleration, which gained consensus among the empirical studies conducted around activity recognition [69,74]. On the other hand, various research studies investigated the impact of combining different other modalities [75,76,77,78,79,80]. The authors in [76], for example, studied activity recognition by using a setting that includes eight sensors: a six-degree-of-freedom accelerometer, microphones sampling 8-bit audio at 16 kHz, IR/visible light, high-frequency light, barometric pressure, humidity, temperature, and compass. In [75], motion sensors (accelerometers, gyroscopes, and magnetic field sensors) have been combined with ultrasonic transmitters in order to track hands for activity recognition in a maintenance scenario.

#### Performance Characteristics of Sensors

The performance characteristics of a sensor are just as (or more) important than its basic function, which is to detect and gauge the phenomenon of interest [12]. In addition to the type of sensing modality, the choice of an appropriate sensing device and its performance characteristics for a given application is one of the most important issues sensor-rich environment designers are faced with. The transfer function defines the relation between the input of the sensing device and its output. Depending on many different factors, sensing characteristics defined by this transfer function may vary substantially. Moreover, depending on the application or the phenomenon being monitored, many different properties are considered with varying importance by the designers, including *span*, *accuracy*, *frequency response*, *sensitivity*, *repeatability*, *resolution*, and *reliability*. Other factors such as costs are also considered. In particular, in the case of mobile computing and applications based on the use of smartphones, the considered sensors are often low-cost, resulting in poor calibration in many occasions, inaccuracies, and limitations in the granularity and range, compared to using dedicated inertial measurement units [72,81,82,82].

The aforementioned aspects are sources of uncertainty when activity recognition models are deployed into real-world sensor-rich environments. Basic settings very often ignore these sources of uncertainty and assume that the model will face ideal deployment scenarios. To better cope with these real-world deployment scenarios, we propose to model these sources of uncertainty via a surrogate (or meta) model, which will act as a proxy and guide smaller models to learn suitable inductive biases and adapt easily to new situations.

## 4. Dynamic Inductive Bias Selection

**Figure 7:**
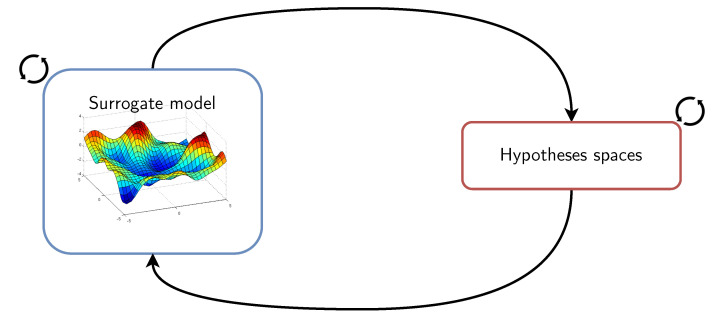
Framework of the proposed approach.

Our framework is framed in a two-levels where a surrogate model or metamodel is used to encode the data acquisition step of the learning pipeline as well as the deployment scenarios. In contrast, simpler models, which are actually deployed, are designed with guidance from the metamodel (Figure 7). These surrogate models have larger capacities in terms of complexity, representativeness, richness, and flexibility in the sense of Vapnik’s definition [83], and, more importantly, involve slower extraction of information. Framed in the multi-level structuring of meta-learning, these (surrogate) models are used to guide smaller models, which, on the contrary, have generally smaller capacity and can be trained rapidly. Conceptually, the idea behind our approach is to remove the barrier that imposes us to fix the inductive biases beforehand (and subsequently the hypothesis space to explore) and instead leverage surrogate models that guide the selection of inductive biases.

### 4.1. Section Organization

In the following, we will first contextualize the components of the known activity recognition chain [16] with regard to the inductive bias learning and the need for selecting them dynamically (Section 4.2). We then present a background on dynamic inductive bias selection [84] and an overview of the long line of research on this paradigm (Section 4.3). Finally, we turn to one instantiation of the dynamic selection of inductive bias paradigm, *surrogate models*. In our use-case, we encode models of the deployments as well as those of the phenomena into surrogate models (Section 4.4).

### 4.2. Background on Supervised Learning

According to the PAC model of machine learning and its variants [85,86,87], supervised learning models typically take the following general form: the learner is supplied with a hypothesis space H and training data $\left\{\left(x_{1},y_{1}\right),\ldots ,\left(x_{m},y_{m}\right)\right\}$ drawn independently according to some underlying distribution *P* on X×Y. Based on the information contained in the training data, the learner’s goal is to select a hypothesis $h:X\overset{}{\to }Y$ from H minimizing some measure $er_{P}\left(h\right)$ of expected loss with respect to *P* (for example, in the case of squared loss $er_{P}\left(h\right):=E_{(x,y)∼P}{\left(h\left(x\right)-y\right)}^{2}$). In such models, the learner’s bias is represented by the choice of H; if H does not contain a good solution to the problem, then, regardless of how much data the learner receives, it cannot learn [84]. In general, models of supervised learning include the following: an input space *X* and an output space *Y*, a probability distribution *P* on X×Y, a loss function $\ell :Y\times Y\overset{}{\to }R$ (empirical risk minimization), and a hypothesis space H which is a set of hypotheses or functions $h:X\overset{}{\to }Y$.

In the case of human activity recognition, one possible mapping is that *X* would be the set of observations generated by the on-body sensor nodes, *Y* would be the set of target activities (walk, run, etc.), and the distribution *P* would be peaked over different episodes during which users perform one of the target activities. The learner’s hypothesis space H would be a class of neural networks mapping the input space *X* to *Y*. The loss in this case would be discrete loss: $\ell \left(y,y^{\prime }\right):=\{\begin{array}{cc} 1 & \mathrm{if}\;y\neq y^{\prime }\\ 0 & \mathrm{if}\;y=y^{\prime }. \end{array}$

**Figure 8:**
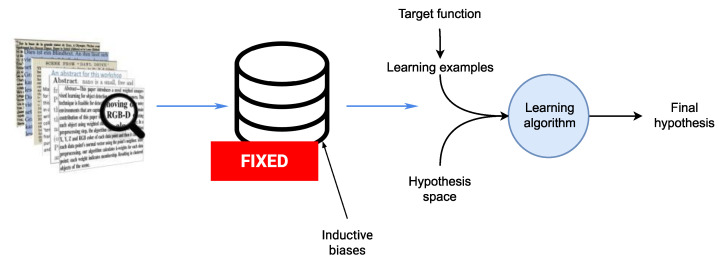
Basic learning setting where the learner is supplied with a fixed set of inductive biases. The inductive biases guide the learner in searching for the hypothesis that explain best the set of learning examples.

Figure 8 illustrates the basic learning setting where the learner is supplied with a fixed set of inductive biases. These inductive biases are the set of all factors that collectively influence hypothesis selection. In the case of human activity recognition from a stream of observations, these factors include for example, the preprocessing, segmentation, feature extraction, and other steps which are part of the activity recognition chain (see Section 2.1). The biases form the ground upon which the learner can choose one hypothesis that explains the examples it sees. In a sense, the biases guide the learner in electing one hypothesis rather than another. Two important features of bias are strength (reduction factor of hypothesis space) and correctness [88]. In addition to the definition of the space of hypothesis and the algorithm that searches for the optimal hypothesis, the learner is supplied with learning examples.

In many real-world situations, however, fixing the biases has a clear disadvantage, particularly when the topology of the sensor deployment evolves or the quality of the sensing nodes are impacted by environmental effects. A trade-off emerges, therefore, between fixing the inductive biases in early stages of the activity recognition models and allowing for a loose specification of these biases, which also has clear disadvantages. One intermediate solution would be to maintain concurrent hypotheses spaces that can be searched for rapidly in order to find the most appropriate hypothesis and inductive biases that apply for the encountered learning configuration.

### 4.3. Background on Dynamic Inductive Bias Selection

**Figure 9:**
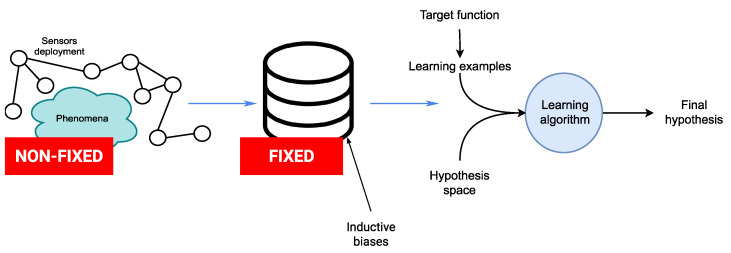
(**left**) Sensor-rich environment depicting a deployment of sensors surrounding a phenomena of interest. (**center**) Data repository where the fixed nature of the inductive biases used during the data generation process is highlighted. (**right**) The different components of the traditional learning processes. Learning processes in the case of sensor-rich environments deal very often with evolving and non-fixed settings materialized by the sensor deployments as well as the phenomenon of interest. This contrasts with the traditional setting where inductive biases are fixed beforehand and assumed to remain relevant during the entire deployment.

As mentioned, sensor-rich environments are characterized by dynamicity. For example, in addition to constant evolution, sensors deployments are often subject to packets loss and heterogeneity, among many other issues. While fixing inductive biases applying to specific problems can be advantageous in controlled environments, performing this operation during early steps of the activity recognition chain in such environments (see Figure 9) inevitably results in inefficient hypothesis space exploration; even worse, the final hypothesis that is elected may fail to explain the learning process. A natural solution is to delay the selection of the inductive biases as late as possible and to maintain concurrent hypotheses which can cope rapidly with new situations. This results in different implications operationally speaking, namely, maintaining a set of alternative inductive bias candidates (the domain) and exploring the space rapidly in order to elect the appropriate hypothesis (amount of supervision with learning examples). In other words, the exploration of the hypothesis space should be structured by leveraging a priori knowledge about the sensor deployments and the phenomenon itself.

In [84], the author proposed a model of bias learning where rather than fixing a unique hypothesis space to search for a satisfactory solution, the learner is supplied with many different hypothesis spaces and, thus, many biases, which could apply to many different problems in the environment. This involves, for the learner, first selecting the most appropriate hypothesis space and then searching for a solution within this space. Thus, in order to enable the learner to learn the bias and select the most appropriate hypothesis space, it is supplied with a family or set of hypothesis spaces H:={H}. Formally, a learning to learn or bias learning problem consists of the following:

- An input space *X* and an output space *Y* (both of which are separable metric spaces);
- A loss function $\ell :Y\times Y\overset{}{\to }R$;
- An environment (P,Q) where P is the set of all probability distributions on X×Y, and *Q* is a distribution on P;
- A hypothesis space family H={H} where each H∈H is a set of functions $h:X\overset{}{\to }Y$.

In the bias learning model proposed in [84], the learner is embedded in an environment of related tasks, e.g., face recognition, character recognition, etc., and, thus, requires fairly dissimilar inductive biases. Here, we rather consider learning configurations that describe the same phenomena (a same task) which evolves itself but also in terms of the sensor deployments used to capture it. More formally, according to the notation in [84] adapted to the problem we are interested in, the set of learning settings that are likely encountered in real-life deployments is represented by a pair (P,Q). P is the set of all probability distributions on X×Y, i.e., P is the set of all possible learning problems or all possible learning scenarios corresponding to a particular configuration of the sensor deployment, and *Q* is a distribution on P. *Q* can control, for example, the various scenarios that the activity recognition model will likely encounter in real-life deployment settings. In the original framework, the distribution *Q* is defined to control the learning problems in the sense of multi-task learning the system is likely to encounter.

The framework that we propose consists precisely in modeling the distribution *Q* via a surrogate model or metamodel, which mimics the behavior of the true distribution as closely as possible while being computationally cheaper to evaluate. In particular, we focus on the data acquisition step and the network of interactions that arise between the sensing nodes of the deployments. Using adequate assumptions, the surrogate model allows us to infer the behavior of the distribution in various situations.

### 4.4. A Surrogate Model for the Data Acquisition Step

**Figure 10:**
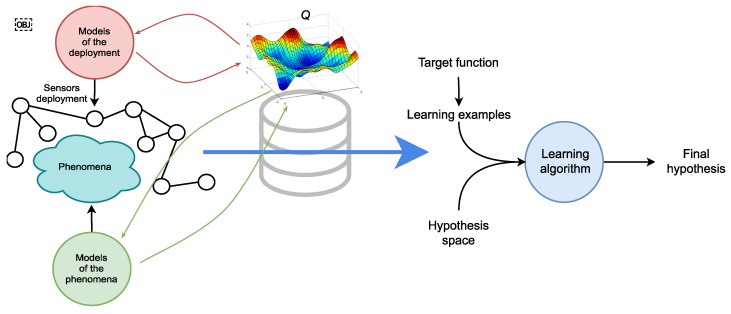
In the proposed framework, we are no longer required to fix the inductive biases beforehand as in the case of the traditional setting. The models describing both the deployment of sensors (in red) and the monitored phenomena itself (in green) serve to guide the learning process by providing the adequate inductive biases dynamically. More formally, the distribution *Q* can control the various scenarios that the activity recognition model will likely encounter in real-life deployment settings, such as the evolution of the deployment topology, the sensing platform, etc. As illustrated, the distribution *Q* can also control the evolution of the monitored phenomena.

With the dynamic nature of sensor-rich environments, we have to delay the selection of the inductive biases as late as possible and maintain concurrent hypotheses that can cope rapidly with new situations or scenarios, and we need models that learn and adapt quickly to new settings, new users, new activities, etc. Figure 10 illustrates this idea. Models of both the deployments as well as those of the monitored phenomena are highlighted. A subsidiary question that we may ask is whether we need to evaluate the activity recognition model on every single scenario that it may encounter during deployment or find other ways to make it adapt rapidly to these scenarios which, we recall, could potentially be encountered by the model for the first time. Evaluating the learning pipeline (in particular, the data acquisition step) for every possible situation according to the distribution *Q* is unfeasible as this distribution could be very complex. Using a surrogate model has the advantage of providing us with a fairly close sense of the true distribution while being computationally feasible. Indeed, under suitable assumptions, sufficient exploration budget, and appropriate sensitivity analysis, the surrogate model can inform us and guide the deployed learning models to better cope dynamically with the environments where they are deployed by using appropriate inductive biases. These metamodels allow us to capture some form of continuity in the space of inductive biases from a reduced number of actually explored instances and extrapolate the properties of the inductive biases to the configurations that the learners will face during actual deployment. This notion of continuity was studied in [89] in the case of neural networks loss function. In the following, we describe the ingredients for constructing surrogate models (Section 4.4.1). In the subsequent sections, we present an instantiation of the proposed framework. In this instantiation, we consider the data acquisition step of the activity recognition pipeline. In particular, we focus on the network of interactions between the sensing devices.

#### 4.4.1. Ingredients of the Surrogate Models

**Figure 11:**
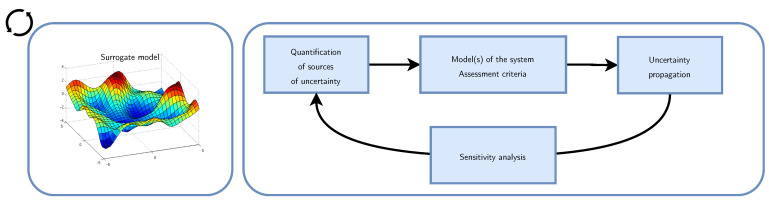
Global framework for uncertainty quantification [91]. This framework is used to model the distribution *Q* controlling the various scenarios that the activity recognition model will likely encounter in real-life deployment settings.

A surrogate model is an approximation of the original computational model M that is computationally cheaper for evaluation and built from a limited set of realizations of the original model. The approximation assumes some regularity of the model and some general functional shape [90]. Figure 11 illustrates the process of surrogate model construction.

The process of surrogate model construction consists of the following components which are executed in sequence [90]:

- Step A consists in defining the model and associated criteria that should be used to assess the system under consideration, which could be a concrete physical system or, in the case of activity recognition pipeline, a cyber-physical system. In our instantiation, we compose architectural components controlled by a set of hyperparameters to encode the activity recognition chain (learning pipeline). The quantity of interest being the recognition performances achieved by the model (see Section 4.4.2).
- Step B consists in identifying and modeling probabilistically the input parameters that brings uncertainty to the system under consideration. We concentrate on the data acquisition and structure of interactions between the sensors of the deployment (see Section 4.4.3).
- Step C consists in evaluating (the response of) the model at specific points of the experimental design space, i.e., concrete realizations of the input parameters that bring uncertainty and identified in Step B. The proposed architectural components and associated hyperparameters are evaluated following a neural architecture search fashion (see Section 4.4.4).
- Sensitivity analysis step consists in analyzing (decomposing) the sources of the model’s response variability with respect to the variation of each individual input parameters. To decompose the sources of variability, we use in our instantiation and Sobol indices (see Section 4.4.5).

#### 4.4.2. Encoding Meta-Knowledge via Neural Architectures

The first step consists in the definition of the model that should be used to assess the aspect of the activity recognition pipeline under consideration. Here, we represent the activity recognition models (or the learning pipelines) under consideration by using neural architectures. The input parameters *k* are the set of hyperparameters of the neural architectures, whereas the outputs ν (or quantity of interest) of the model are the recognition performances of the neural architecture. Note that the notion of recognition performances is linked to the data used for training and validation. However, one can evaluate an architecture directly without performing a training phase, given that the bias related to the architecture’s structure tailors specifically the target task (see, e.g., weight-agnostic neural architectures [92]).

**Figure 12:**
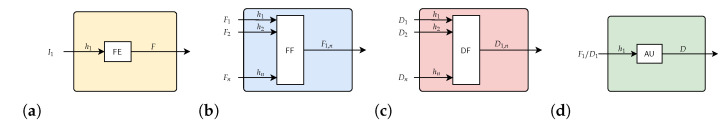
Architectural components used to construct the neural architectures. (**a**) Feature extraction (FE), (**b**) feature fusion (FF), (**c**) decision fusion (DF), and (**d**) analysis unit (AU).

**Figure 13:**
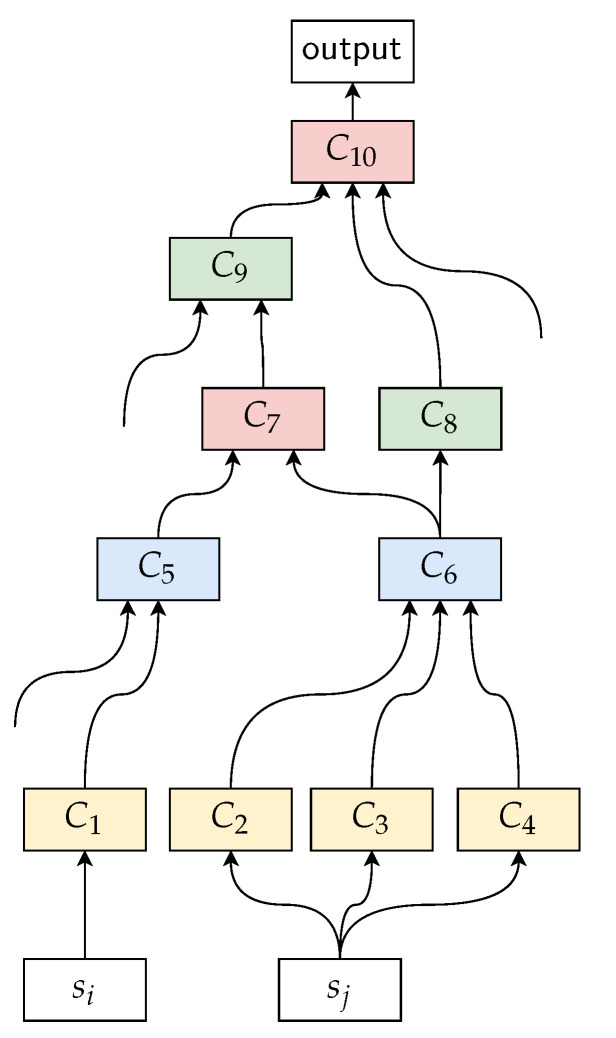
An example of architecture constructed using the architectural components depicted in Figure 12. The nodes $s_{i}$ and $s_{j}$ correspond to the data sources.

Figure 12 illustrates the four types of architectural components, including *feature extraction* (FE), *feature fusion* (FF), *decision fusion* (DF), and *analysis unit* (AU), that we use to construct neural architectures. These components process their inputs, which can be raw data, features, or decisions, and outputs including either features or decisions. The process performed by these components can involve preprocessing steps, feature extraction, raw data or feature fusion, decision fusion, etc. Additionally, we define, for each individual input, a hyperparameter that controls how the component processes that input and subsequently its influence on the final performance achieved by the overall architecture that is constructed by combining these components. The constructed architectures are represented as a directed acyclic graph where the architectural components form the vertices and the set of directed edges that connects them together. Each edge of the graph is assigned with a value $h_{u}^{v}$, which corresponds to the hyperparameter that controls how the associated component processes the data flowing into it. The set of all hyperparameters of a given architecture is referred to as H (Figure 13 illustrates an example of a constructed architecture). An instantiation (or a realization) of the set of hyperparameters with actual values for each of them produces a particular architecture, indexed as *k*.

Internally, the sampled architectures (or the realizations of the set of hyperparameters H) are trained on real data, e.g., in the case of human activity recognition, the architectures are fed with pairs consisting of motion data and labeled activity. Training is framed as a sequence classification problem, where the goal is to learn a function $F:X\overset{}{\to }Y$ mapping inputs to outputs. Note that, here, the inputs to the architecture are not the same as the input to the model used to represent the learning pipeline under consideration. As in the traditional classification setting, performance of the neural architecture is quantified with a loss function $\ell :X\times Y\overset{}{\to }R$, and a mapping is found via the following:

(1) $$\begin{array}{c} f^{*}=\underset{f\in F}{\mathrm{argmin}}\frac{1}{N}\sum_{j=1}^{N}\ell \left(f\left(x_{j}\right),y_{j}\right) \end{array}$$

which can be optimized by using a gradient descent algorithm over a pre-defined class of functions F. In the example of convolutional layers provided above, F can be convolutional networks parametrized by their weights and the loss function can be $\ell \left(f\left(x_{i}\right),y_{i}\right)=1\left\{f\left(x_{i}\right)\neq y_{i}\right\}$. For a particular instantiation of the hyperparameters, the weights of the resulting architecture will be tuned during the optimization process.

#### 4.4.3. Data Acquisition and Structure of Interactions between Sensors

The aspect of the learning pipeline that we are trying to represent here is the data acquisition step which involves quantifying the uncertainty that stems from the individual data sources as well as their interactions. These are the sources of uncertainty to our model and that are part of the second step of the metamodeling process.

The data acquisition step is tightly linked to the dynamics of the body movements. These dynamics are an important a priori knowledge that is often considered in activity recognition models, e.g., [93,94,95,96,97]. To represent the data acquisition step in relation with the neural architectures we defined in the previous section, we propose to encode two notions: the *importance* of a data source and the degree of *interaction* between a set of data sources. Let $s_{i}$ be a data source attached to a given body part and *y* is an activity that we want to recognize. The importance of $s_{i}$ with regards to activity *y*, denoted $\mu_{i}^{y}\in \left[0,1\right)$, is defined as a quantity that represents the relative involvement of that body part in the dynamics of the gestures pertaining to that activity. On the other hand, an interaction between a set of data sources S∈S, denoted $\mu_{S}^{y}\in \left[0,1\right)$, is defined as their level of dependence with regards to the relative involvement of the body parts they are attached to in the dynamics of the gestures.

To link these two notions with the neural architectures and their associated hyperparameters defined above, we consider the set of hyperparameters, denoted $H_{s}⊊H$, which control how a given data source *s* is processed by an architecture to be representative of the global impact (or importance) of that data source. The main principle is that the structure of the architecture, which is determined by its associated hyperparameters, is critical, and finding the right instantiation of hyperparameters can result in an optimal exploitation of the sensory inputs.

In order to quantify these two notions, we have, first, to determine the correspondence between the data sources and the hyperparameters associated with the proposed architectural components. Indeed, we have access only to the values associated with the hyperparameters and not the data sources directly. Let *A* be an architecture, the correspondence between each individual data source and the hyperparameters associated with the architecture *A*, denoted $Corr_{A}:S\overset{}{\to }℘\left(H\times R\right)$, is defined as follows:

(2) $$\begin{array}{c} Corr_{A}\left(s\right)=\underset{\left(u,v\right)\in s{\overset{}{\to }}^{*}t}{⋃}<h_{u}^{v},w>, \end{array}$$

where

- $s{\overset{}{\to }}^{*}t$ is a subset of all the paths in the architecture that have *s* as a source and *t* as a sink;
- $h_{u}^{v}$ denotes the hyperparameter associated with the edge (u,v);
- $w=\frac{\omega_{1}\cdot dist\left(s,v\right)+\omega_{2}\cdot \delta^{-}\left(v\right)}{\omega_{1}+\omega_{2}}$ is a weight that ponders the impact of the hyperparameter $h_{u}^{v}$ according to how far is it from the input and how many edges goes into the component *v*.

**Figure 14:**
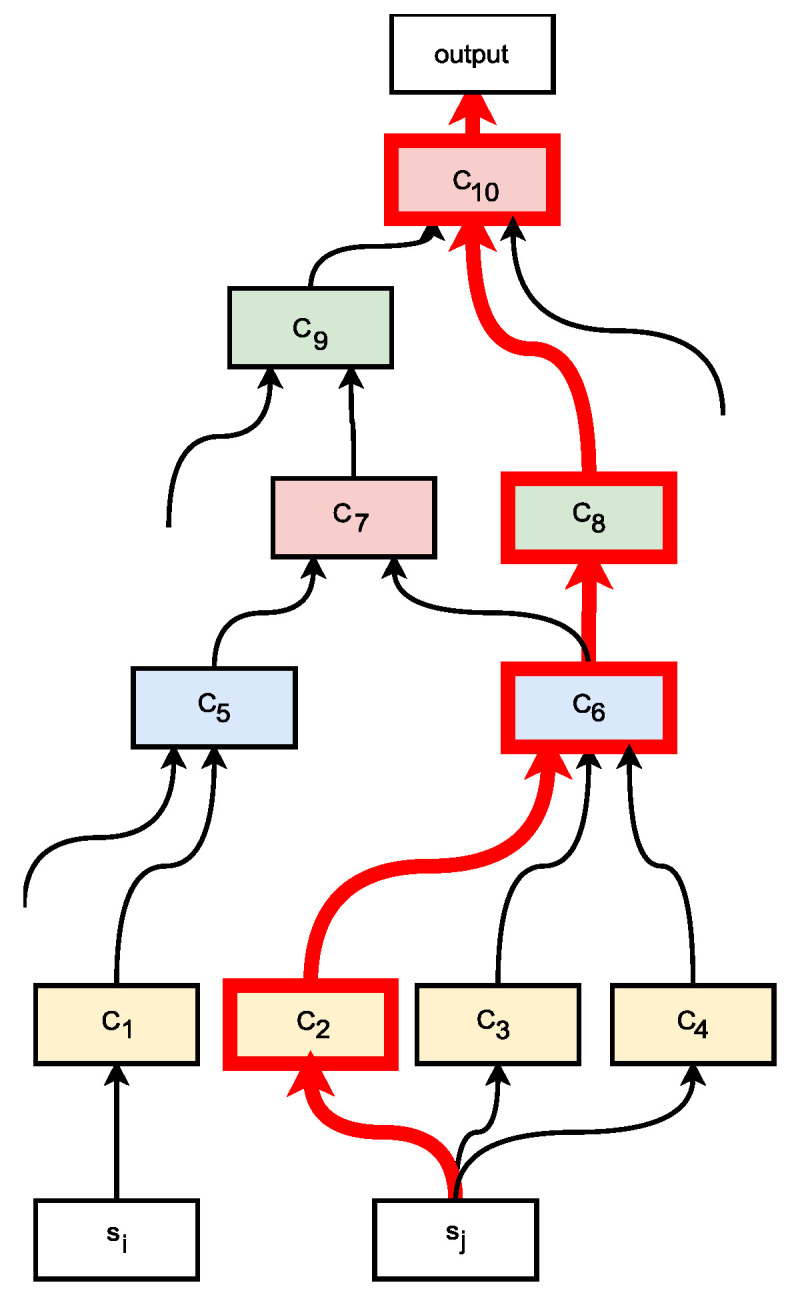
Highlighted in red is one of the paths that have as source node the data source denoted by $s_{j}$ and that is processed by the architectural components $C_{2}$, $C_{6}$, $C_{8}$, and $C_{10}$ before joining the architecture’s output node.

Note that if an edge is included in more than one path, the weights assigned to the corresponding hyperparameter following each path are summed. Figure 14 illustrates one of the paths, highlighted in red, that have *s* as a source and proceeds throughout the architecture to the output.

#### 4.4.4. Uncertainty Propagation via Architecture Search

After defining the sources of uncertainty to our model, the third step consists in propagating the uncertainty of the input variables to the model’s response. This step corresponds to evaluating the model’s response for multiple realizations of the input variables; in our case, this is the set of hyperparameters which in turn involve specific data sources of the deployment. More formally, let $K=\{k_{1},k_{2},\ldots \}$ be a set of instantiations of the considered hyperparameters. For each instantiation, the quantity of interest, i.e., the recognition performances of the resulting architecture, is evaluated and yields ultimately the response surface $\{M\left(k_{1}\right),M\left(k_{2}\right),\ldots$}. The weights associated with a given architecture are obtained by optimizing the individual weights of the architectural components using, for example, a gradient descent algorithm over a predefined class of functions.

**Figure 15:**
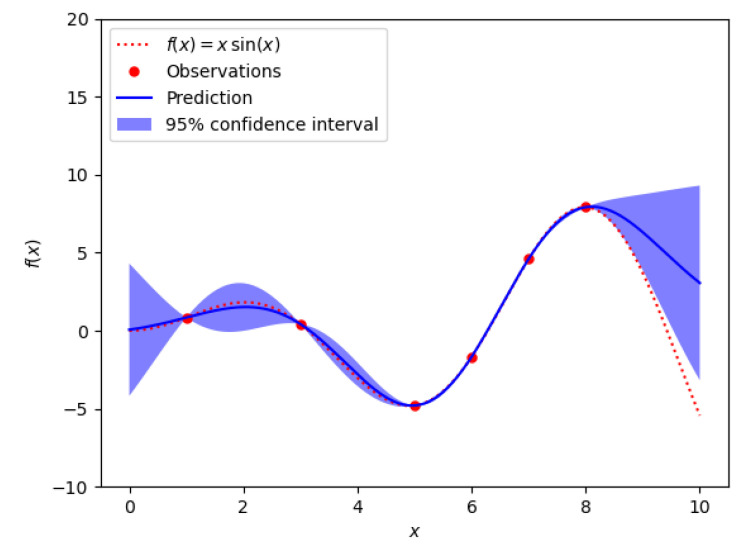
An example of a Kriging (also known as Gaussian process modeling) metamodel The observations correspond to the actual evaluations of the hyperparameter instantiations. Kriging assumes that the true model is a trajectory of an underlying Gaussian process. The x-axis represents the design space (experimental points or realizations) while the y-axis represents the model’s response.

It is worth noting that one can make use of a predefined set of experimental points (or realizations) carefully designed to capture the model’s response. An alternative method is to use adaptive experimental designs where one starts from a small set of experimental points and enrich it with new points in suitable regions. After evaluating the model’s response at a particular point of the experimental design space, another point has to be picked where, again, the model will be evaluated. This process is repeated until the allocated budget is exhausted. The way these points are picked is determined by the assumed underlying model trajectory. For example, Kriging [98] (or Gaussian process modeling) is suitable for adaptive experimental designs. Kriging assumes that M(k) is a trajectory of an underlying Gaussian process $M\left(k\right)\approx \beta^{⊤}f\left(x\right)+\sigma^{2}Z\left(x,\omega \right)$, where *Z* is a zero mean unit variance Gaussian process and the parameters {ω, β, and $\sigma^{2}$} are estimated from the experimental design by maximum likelihood estimation, cross validation or Bayesian calibration (see Figure 15).

In our case, this outer optimization loop (which should be contrasted with the inner optimization loop of the model’s weights) handles pairs of hyperparameters instantiation, *k*, and global final performances, ν=M(k). This outer process turns out to be what is referred to as neural architecture search. The problem of modeling the data acquisition step is then cast as the exploration of the architecture space which is usually determined by a search space, a performance estimation strategy, and a search strategy [99]. The search space is defined by the type of architectural components (similar to those defined in Section 4.4.3) and the way these are connected together (e.g., unique vs. multiple branching). The performance estimation strategy can be as simple as the classification accuracy achieved by the architecture in a sequence classification problem, while the search strategy is the process that decides which points (or regions) of the architecture space should be evaluated. This last aspect is what determines the new design points (or realizations) to pick, possibly in suitable regions of high expected reward. More formally, the selected exploration strategy tries to find an architecture $k^{*}$ that maximizes the recognition performances (or minimizes the validation loss) $\nu_{k^{*}}\left(w^{*}\right)$. Given an exploration budget *B*, the exploration strategy yields a series of the model’s responses (recognition performances or validation losses) $\left\{M\left(k_{1}\right),M\left(k_{2}\right),\ldots ,M\left(k_{B}\right)\right\}$ (or $\left\{\nu_{1},\nu_{2}\ldots ,\nu_{B}\right\}$). The task of uncertainty quantification, therefore, reduces to decomposing these model’s responses into the impact of each individual data source.

#### 4.4.5. Variance-Based Importance Estimation

Sensitivity analysis is defined as the process aiming at quantifying which input parameter (or combinations thereof), in our case the data sources featured by sensor deployments, influences the response variability of the model the most [90]. Given the set of validation losses (or model’s responses) obtained from the previous step, we estimate the importance of each data source by decomposing the variation of the non-linear relation M into an additive expansion (or Sobol indices) due to each of its inputs [100]. It is defined as follows:

(3) $$M\left(S,y\right)=\mu_{0}^{y}+\sum_{i=1}^{M}\mu_{i}^{y}\left(s_{i}\right)+\sum_{i\neq j}\mu_{ij}^{y}\left(s_{i},s_{j}\right)+\ldots +\mu_{1\ldots M}^{y}\left(s_{i},\ldots ,s_{M}\right),$$

where $\mu_{0}^{y}$ is a constant mean, $\mu_{i}^{y}$ the first-order effects, $\mu_{ij}^{y}$ the second-order effects, etc. The lower the variance induced by a data source, the higher its influence on the non-linear relation M.

The proposed surrogate model allows us to model the uncertainty of the data acquisition step. The modeled uncertainty or meta-knowledge will be leveraged in order to guide the exploration of the hypothesis space and the selection of the most suitable set of inductive biases.

### 4.5. Surrogate Model-Informed Selection of Inductive Biases

Here, we focus on the incorporation of learned (optimized) meta-knowledge in the sense of the Vapnik and Hinton frameworks, i.e., high-capacity surrogate models that supervise simpler (lower-capacity) models in order to accelerate their learning and adaptation. Since IoT environments are characterized by evolutivity and dynamicity, the presence of constraints related to the nodes requires much lighter models supervised with more flexible boundaries between classes, etc.

**Figure 16:**
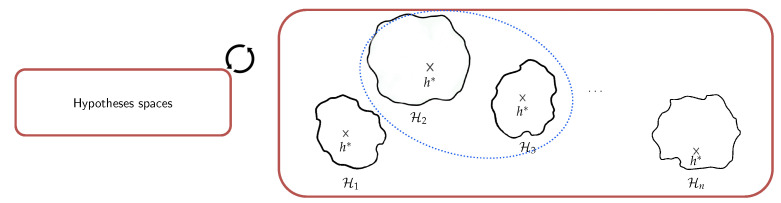
Surrogate model-informed selection of inductive biases where, among the hypothesis spaces family H, a set of concurrent hypothesis spaces (each of which containing a possibly suitable optimal hypothesis) is maintained throughout the learning process and model deployment.

Figure 16 illustrates the notion of selection of inductive biases which can be regarded as keeping a meta-hypothesis that has the ability to be adapted rapidly to new configurations. Here, the surrogate model can be leveraged in order to structure the family of hypothesis spaces that allows easy traversal (or navigation) between them. The incorporation of the derived knowledge with regards to the selection of inductive biases (or hypothesis spaces) can take many forms, including supervision with privileged information [101] and knowledge distillation [102] ( Note that various works have been pursued to unify these two frameworks into a more general one, e.g., [103]).

In the case of the privileged information framework, Vapnik et al. in [101] make an analogy with the fact that humans learn much faster than machines and illustrate this with the Japanese proverb *“better than a thousand days of diligent study is one day with a great teacher”*. The proposed *learning with privileged information* framework consists in considering training data formed by a collection of triplets $\left\{\left(x_{1},x_{1}^{*},y_{1}\right),\ldots ,\left(x_{n},x_{n}^{*},y_{n}\right)\right\}∼P_{n}\left(x,x^{*},y\right)$, where each $\left(x_{i},y_{i}\right)$ is a feature-label pair, and the privileged information $x_{i}^{*}$ is an additional supervision term about the example $\left(x_{i},y_{i}\right)$ provided by an intelligent teacher (in our case, the surrogate model) in order to support and guide the learning process. Here, guiding the learning process can either be linked to the learning examples supplied to the learner or the learning configurations (e.g., the topology of the sensor deployments, characteristics of the sensing devices, etc.) that the learner encounters during deployment. The privileged information can be, e.g., relevant features or sample-dependent relevant features [103]. The selection of the suitable hypothesis spaces can leverage the uncertainty accompanying some configurations of the data acquisition step.

On the other hand, the distillation framework introduced in [102] tries to incorporate knowledge, in the form of class-probability predictions, from high-capacity models into low-capacity models. Rather than training low-capacity, deployment-ready models using the raw (hard) labels, class-probability predictions (soft labels) generated by the high-capacity models are used instead. In contrast to a boosting training strategy where the hard-to-classify examples are weighted so that the learner can focus on them, in this framework, the easy-to-classify examples, in the sense of smooth class membership, are supplied during model training instead. This smoothness in class membership (or class probability predictions) is controlled by using an additional parameter (temperature ∈]0,1[) which decides how to soften the class membership.

In our approach, we leverage the dynamics of the body movements and the fact that each activity is defined by a specific set of gestures which in turn involves specific body parts equipped with data sources. We train simpler models by selecting subsets of data sources that are highly confident and informative regarding these dynamics in order to create a curated training set for model training. In this manner, the constructed models will be able to cope with the evolution of the sensor deployments.

## 5. Case Study: Shl Dataset Deployment

**Figure 17:**
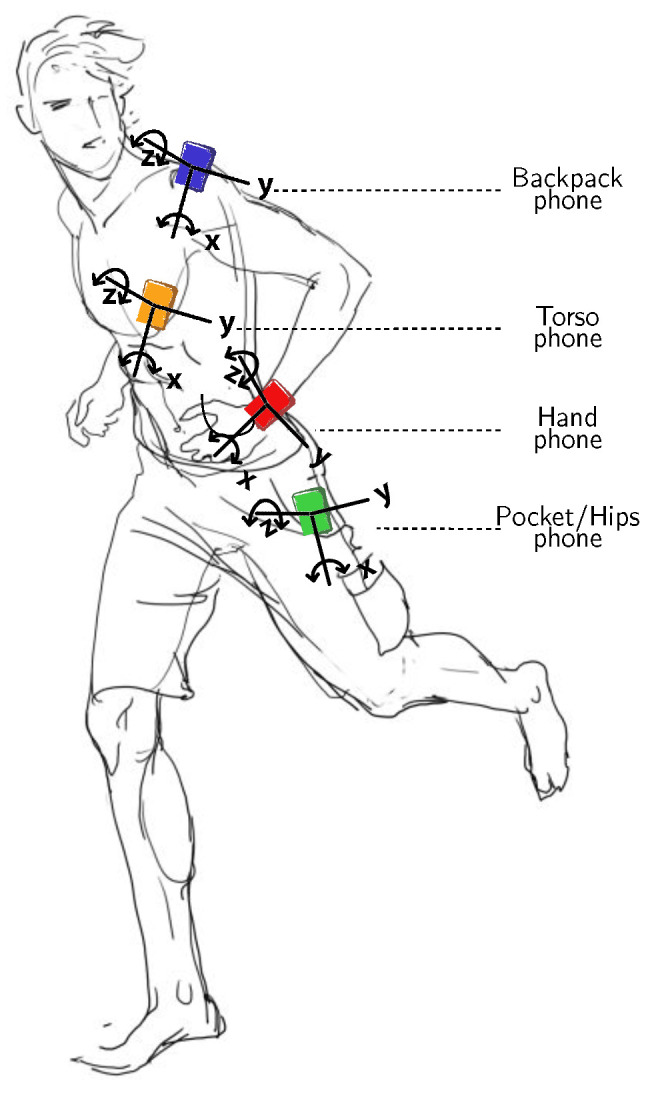
Topology of the on-body sensors deployment featured by the SHL dataset, which is used in the experimental part of this paper. Data collection was performed by each participant using four smartphones simultaneously placed in different body locations: *Hand*, *Torso*, *Hips*, and *Bag*.

Here, we describe the SHL dataset, which is the main dataset used in our empirical evaluation [18] (the preview of the SHL dataset can be downloaded from: http://www.shl-dataset.org/download/ (accessed on 30 October 2021)).We chose to experiment primarily on this dataset as it features multi-modal data generated from sources located on various body locations and recorded in real-life settings over a period of 7 months in the United Kingdom. Among the 16 modalities of the dataset, we focus in this study on the body-motion modalities including accelerometer, gyroscope, magnetometer, linear acceleration, orientation, gravity, and ambient pressure in addition. Data were collected from a set of four sensor-enabled smartphones, all of which are synchronized and function simultaneously. Each smartphone is placed on one of the following body locations: *Hand*, *Torso*, *Hips*, and *Bag*. These four positions define the topology of the sensors deployment that allows capturing the dynamics of the body movements. In total, eight activities are considered in the dataset including *Still*, *Walk*, *Run*, *Bike*, *Car*, *Bus*, *Train*, and *Subway*. Figure 17 shows the on-body sensors deployment used during data collection. Note that we make use in our experiments of other datasets, namely, USC-HAD [20], HTC-TMD [21], and US-TMD [22], which are described in more detail in Section 6.3.

## 6. Evaluations

We conduct in this section an empirical evaluation of the dynamic inductive bias selection via two main axes: (i) analysis of a surrogate model based on Gaussian processes (Section 6.2) and (ii) incorporation of the derived privileged information via adaptive sampling (Section 6.3). For reference, we evaluate a basic activity recognition chain which features a set of fixed set of inductive biases (Section 6.1).

### 6.1. Basic Activity Recognition Chain

In this first set of evaluations, we consider a basic activity recognition chain. Here, the basic activity recognition chain illustrates the effects of fixing the inductive biases in advance on recognition performances.

For this set of experiments, we constructed the baseline activity recognition chain by using neural network-based layers. Neural networks are often used in the context of human activity recognition yielding good performances in general, e.g., [78,104,105,106] which is primarily due to their ability to efficiently aggregate heterogeneous data which is the case in activity recognition from wearable sensors. For us, the principle is to model the entire activity recognition chain including each of its constituting steps (described in Section 2.1) as a unique neural architecture that global behavior will emulate the work conducted by every single step. Indeed, neural network, and convolutional-based layers, in particular, have the advantage of learning in an automatic fashion the hierarchies of abstract features as well as adapted processes that fit our modeling needs (e.g., setting the suitable segmentation parameters and the right combination of features capable of separating the different activities correctly). This would be particularly advantageous for modeling the data acquisition step and capturing the uncertainty that stems from the sensor-rich environment featured by the SHL dataset.

More precisely, the basic activity recognition chain is constructed by stacking Conv1d /ReLU/MaxPool blocks. The output layer is formed by full connected (or dense) layers. Regarding the input layer, we define three convolutional modes: grouped modalities, split modalities, and split channels. These modes determine how the inputs are being processed by the architecture’s front-end. Regarding the input signals, these are taken as they are without any additional segmentation process other than the one induced by the convolutional layers. The inputs to the activity recognition chain are, therefore, of the order of 1 min, i.e., 6000 time-steps, given a sampling rate of 100 Hz.

**Figure 18:**
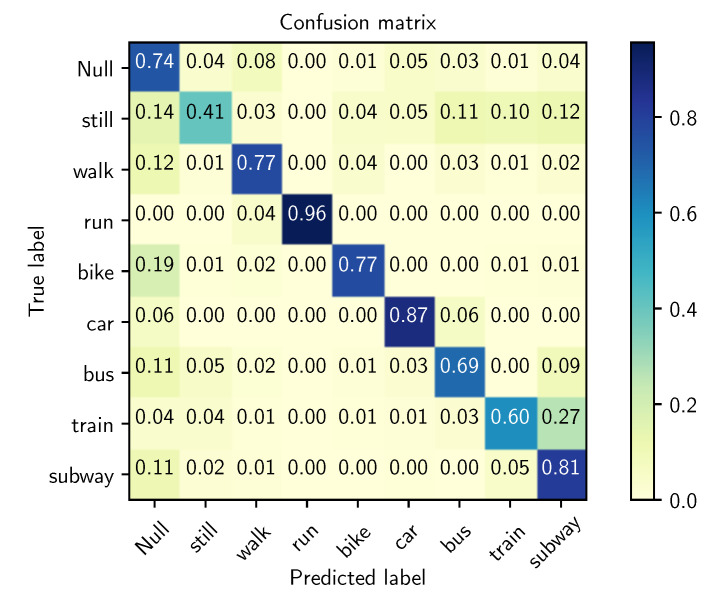
Confusion matrix of the best model trained on all body locations fused together yielding 70.86% recognition performance measured by the f1-score. Best model among the set of models evaluated as part of a Bayesian optimization process involving the hyperparameters of the activity recognition chain.

Figure 18 shows the confusion matrix of the basic activity recognition chain. The overall recognition performance of this model is 70.86% measured by the f1-score. Note that this score corresponds to the best model obtained after performing an optimization of its hyperparameters. Additionally, as no privileged information is incorporated into this baseline, data generated from each one of the data sources featured in the SHL dataset and those we consider are used to train the model. In the following, these results will be used as reference for comparison to assess the proposed approach.

### 6.2. Surrogate Model Based on Gaussian Processes

In this set of experiments, we detail the construction of the surrogate model for the data acquisition step. We first provide the experimental setup including the architectural components (and their hyperparameters) used to represent the activity recognition chain. The surrogate model’s response surface is then analyzed following different levels of granularity (realizations, hyperparameters, and data sources). The experimental setup is presented in more detail in [24].

#### 6.2.1. Experimental Setup

In Section 4.4.2, these architectural components were described in a high-level fashion. Here, we provide concrete instantiations of the architectural components and their accompanying hyperparameters. We use similar architectural constructions as those used for the baseline activity recognition in Section 6.1, i.e., the set of Conv1d/ReLU/MaxPool blocks along with the three convolutional modes. As explained in Section 4.4.2, the architectural components are parameterized by hyperparameters that control how they process their inputs. The blocks we use here are parameterized by the number of filters (or kernels) ($n_{f}$) they encompass as well as their respective sizes (ks, for kernel size). These, among others, form the set of hyperparameters being directly involved in the process of uncertainty quantification. Additionally, we experiment with two types of output layers: fully connected (or dense) and recurrent layers. The fully connected layers are parameterized by the number of units ($n_{u}$) they encompass, while the recurrent, precisely LSTMs, layers are parameterized by the number of hidden units ($n_{hu}$). Of course, depending on the depth at which the block (or layer) is positioned within the neural architecture, we use an integer subscript that corresponds to the depth. We use a sequential (pre-defined) stacking strategy to place the various architectural components ( note that more complex stacking and branching strategies are available for constructing the neural architectures [107,108]). Table 1 provides a summary of the architectural components’ hyperparameters and their respective ranges that were explored in our experimental evaluations.

In the proposed framework, the uncertainty propagation step is performed via a neural architecture search where the experimental points (or concrete realizations) are discovered progressively. Depending on the search strategy used to pick these experimental points, the final surrogate model will result in different privileged information. In order to investigate this aspect, we instantiate the uncertainty propagation step using various exploration strategies. Various tools exists in the literature that provide a comprehensive list of exploration strategies. Among these tools, Microsoft-NNI (Neural Network Intelligence) (https://github.com/microsoft/nni (accessed on 30 October 2021)) constitutes one of the most complete tool. In the following, we enumerate the exploration strategies being investigated and that are organized into their respective categories:

(1) Exhaustive search:
  – Random search [110];
  – Grid search [110];
(2) Heuristic search:
  – Naive evolution [111];
  – Anneal [112];
  – Hyperband [113];
(3) Sequential model-based optimization:
  – Bayesian optimization hyperband [114];
  – Tree-structured Parzen estimator [112];
  – Gaussian process tuner [112].

Concerning the uncertainty quantification step, the decomposition of the non-linear relation defined by the surrogate model can be computed by using the efficient implementation proposed in [115], which is based on a linear-time algorithm for computing the marginals of random forest predictions. The visualizations of the structure of interactions between data sources are produced by using the fanova-graph [116].

#### 6.2.2. Analysis of the Surrogate Model’s Response Surface

In this part, we (1) perform an analysis of the low-level aspects of the surrogate model’s response surface related directly to the hyperparameters being optimized at each layer of the neural architectures; (2) we move to the higher levels of the analysis where we focus on the most important and interacting data sources which capture ultimately the network of interactions and the uncertainty accompanying the data acquisition step.

**Figure 19:**
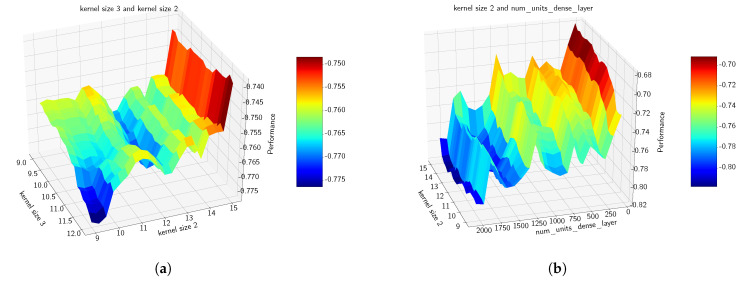
Pairwise marginal plots produced via the fANOVA framework [115]. These plots illustrate the interplay (or the mutual impact influence) between some of the hyperparameters being optimized in (**a**) interplay between kernel sizes 2 ($ks_{2}$) and ($ks_{3}$) (**b**) interplay between the number of units of the dense layer ($n_{u}$) and kernel size 2 ($ks_{2}$).

Table 2 provides a summary of the hyperparameters’ importance obtained using the fANOVA analysis of the model’s responses at specific realizations of the experimental design space. Figure 19 illustrates the pairwise marginal plots of a set of hyperparameters obtained also via the fANOVA framework.

**Figure 20:**
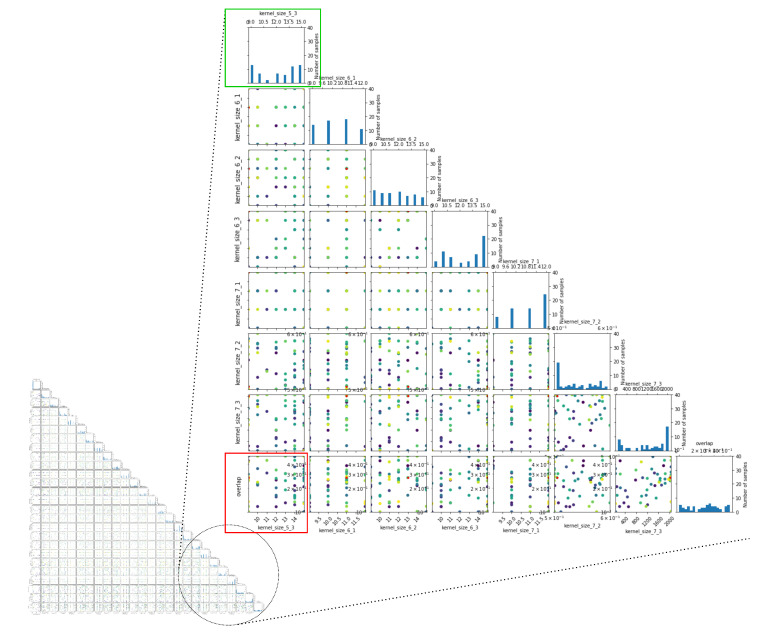
Summary of the hyperparameters instantiations (or realizations) that have been evaluated during the construction of the surrogate model. For example, the highlighted plot (red box) shows the joint instantiations of the hyperparameter overlap (or stride) (*s*) and the kernel size ($ks_{5}$). The realizations are illustrated with graduated colors representing the quantity of interest (recognition performance) of the model at this location of the space. The figure zooms on the rightmost part of the diagram for better readability. The plots on the diagonal show the distribution of the realizations for each individual hyperparameter. For example, the highlighted plot (green box) describes the distribution of the instantiations in the case of kernel size ($ks_{5}$).

Figure 20 illustrates the realizations that have been evaluated during the construction of the surrogate model. It highlights in particular the distribution of the experimental design points (or realizations) that are explored throughout the construction of the surrogate model: some realizations are sparse while others are spread and span all over the space of experimental designs.

**Figure 21:**
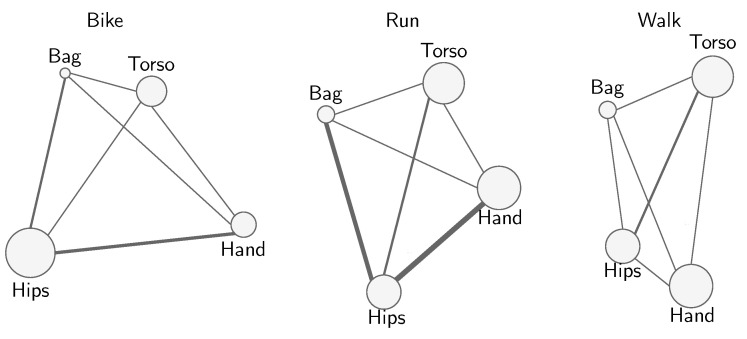
Estimated interaction structure of the data sources for 3 different activities (bike, run, and walk), using the fANOVA graph [116]. Data sources are grouped by their respective positions. The circumference of the circles represents main effects (importance), and the thickness of the edges represents total interaction effects. From [23].

While the discussion above was related to the behavior of the individual sets of hyperparameters, here we provide some high-level insights linked directly to the data sources. Figure 21 illustrates the estimated interaction structure (or fANOVA graph [116]) of the data sources for three different activities and highlights the most important and interacting data sources with circles with larger circumference. The data sources located on the hips are overall more informative in the case of a large number of activities, while in the case of the activities involving “bus” and “run”, the data sources located on the hips yield more important variability and, thus, more pronounced uncertainty. In the case of the activities “walk”, “bike”, and “car”, these same data sources seem to provide sustainable elements to recognize these activities. The prominence of the data sources located on the hips regarding the recognition of human activities is confirmed by empirical results obtained in various studies [22].

Here, we investigate the effects of the space exploration strategy used to determine the experimental points of the space to be evaluated. We take a look specifically at the derived knowledge and to what extent it differs from the human expertise aggregated into what we refer to as human expertise-based model (HExp). To assess this, we use Cohen’s kappa coefficient [117]. This coefficient is often used to measure the level of agreement between two experts or raters. Additionally, we investigate the partial recognition performances obtained while exploring the space, i.e., training and evaluation of the selected architectures. This can be a good indicator of, for example, the concentration of good performing architectures in some regions of the space and, thus, much more exploitable knowledge.

The obtained results using the different exploration strategies listed in Section 6.2.1 are summarized in Table 3. We can see that, in terms of the categories of exploration strategies, the derived knowledge from the sequential model-based strategies agree more with human expertise than their heuristic search-derived counterpart. Knowledge derived using the search-based strategies is the one that agrees the least with human expertise, with a level of agreement measured by Cohen’s kappa coefficient that is less than 0.3, even with a larger exploration budget. Among the sequential model-based strategies, the GP tuner allows us to derive knowledge with the highest level of agreement with human expertise; as such, we will focus, in the following, primarily on it in order to investigate the effectiveness of incorporating privileged information into the activity recognition models.

### 6.3. Incorporation via Sample Selection

Given the surrogate model, the task now is to incorporate the derived knowledge into low-capacity, data-efficient, and deployment-ready models. Here, we describe the experimental setup used to incorporate the dynamics of body movements derived from the surrogate model. The SHL dataset is used to derive the surrogate model of the data acquisition step. We incorporate the derived information into the activity recognition models constructed with the SHL dataset and three additional datasets including USC-HAD [20], HTC-TMD [21], and US-TMD [22]. Table 4 provides some important details about these datasets.

In the following experiment, we incorporate the knowledge derived from the surrogate model into the deployed activity recognition models by selecting the appropriate combination of data sources for each individual activity. Indeed, the dynamics of the gestures, which involve particular parts of the body, characterize to a large extent the activities we are interested in. When the data sources are attached to these body parts, their contribution to the recognition of a given activity is proportional to the involvement of the body parts these are attached to. This is why we ponder the data according to the data source it originates from. More precisely, the knowledge (respective importance and interactions) derived from the surrogate model is used as an indicator function that determines which data sources are highly confident and informative with respect to a given activity. The data generated from these sources will form the training sets used to train the activity recognition models. Formally, for a given activity y∈Y, the set of highly confident and informative data sources $S_{y}$ is defined as follows:

(4) $$\begin{array}{c} S_{y}=\left\{s_{i}\in S|M\left(\left\{s_{i}\right\},y\right)=\mu_{s_{i}}^{y}\geq \tau_{imp}\right\}\cup \left\{S⊊S|M\left(S,y\right)=\mu_{S}^{y}\geq \tau_{int}\right\}, \end{array}$$

where $\tau_{int}$ and $\tau_{imp}$ (int for interaction and imp for importance) are thresholds above which a given set of data sources S⊂S is considered to by highly confident and informative. In particular, for $\tau_{imp}$ = $\tau_{int}=0$, $S_{y}=S$, i.e., the data generated from every single data source are considered in the training set.

As stated previously, the deployed activity recognition models are of much lower capacity in order to comply with the various constraints surrounding the actual nodes where the models will be deployed (see Section 7 for a deeper discussion on these aspects). The deployment-ready activity recognition models we consider in the following are based on convolution layers, i.e., Conv1d/ReLU/MaxPool blocks, but restricted to only three layers followed by a Fully Connected/ReLU layers.

Using the constructed training set which is fed into the activity recognition models, we constrain the models to concentrate on highly informative data sources; subsequently, they are insensitive to the uninformative ones. The constraints are specified via sample-dependent relevant data sources. Training data are formed by a collection of triplets $\left(x_{i},x_{i}^{*},y_{i}\right)$ sampled as follows: $x_{i}\leftarrow x_{i,x_{i}^{*}}$, $x_{i}^{*}\leftarrow S_{y_{i}}$, and $y_{i}\leftarrow y_{i}$. The sets of data sources $S_{i}$ are a subset of the collection of sensors $S=\{s_{1},\ldots ,s_{M}\}$ derived from the surrogate model previously constructed. The input vector $x_{i,x_{i}^{*}}$ contains the relevant data sources $S_{y_{i}}$ which depends on the corresponding activity $y_{i}$ assigned to the original sample $x_{i}$. The remaining parts of the input vector $x_{i,x_{i}^{*}}$, i.e., the unimportant data sources, are assigned values drawn from a normal distribution. The privileged setting is referred to as *w*-prvlg. For a matter of comparison, we make use of training sets constructed by using all data sources to train activity recognition models, i.e., without incorporation of privileged information from the surrogate model nor from human expertise. These models constitute our baselines, and we refer to this setting as *wo*-prvlg. In addition, we compare the impact of incorporating privileged information originating from human expertise (HExp). This setting is referred to as *w*-HExp. Table 5 compares the recognition performances obtained, on each dataset, using these settings.

Overall, incorporation of privileged information either derived from the surrogate model or human expertise allowed us to obtain substantial improvements for all the considered datasets (e.g., 70.86% ± 0.12 $\overset{}{\to }$ 88.7% ± 0.6 in the case of the SHL dataset).

**Figure 22:**
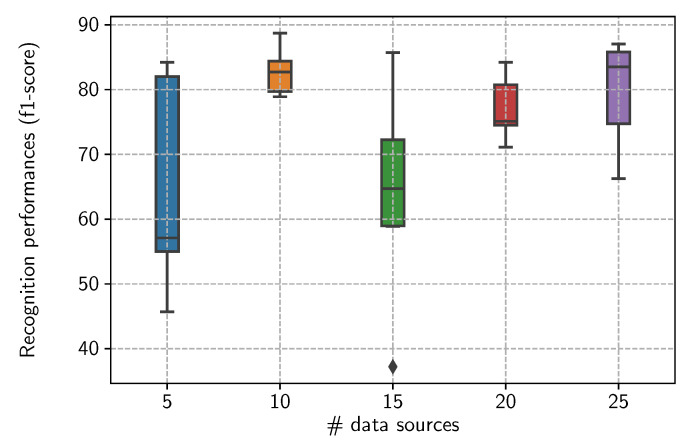
Recognition performances, measured by the f1-score, as a function of the number of data sources (the cardinality on average of the subsets $|S_{y}|$) used to train the deployed activity recognition models. The configuration with 25 data sources corresponds to subsets where all data sources are used, i.e., no privileged information provided.

Becoming closer to the derived privileged information and the subsets of highly informative data sources, Figure 22 illustrates how the number of data sources impacts the performances of the activity recognition models. As the direct action of incorporating the privileged information is to shrink (depending on the thresholds $\tau_{int}$ and $\tau_{imp}$ defined earlier) the number of data sources considered during the training phase, here we assess the obtained recognition performances as we vary the two thresholds and consequently the number of considered data sources. Overall, the activity recognition models trained on smaller subsets of data sources outperform the baseline counterpart which we recall is trained on all the data sources featured by the SHL dataset. Noticeably, the best recognition score, measured by the f1-score, obtained in this set of experiments was 88.7% ± 0.6. More importantly, this score is obtained by using a subset containing solely 12 data sources on average. This constitutes an improvement of approximately 17% compared to the baseline using half of the available data sources.

Additionally, even in the interval between 5 and 15 data sources, we still obtain good recognition rates while in some configurations (e.g., $|S_{y}|=13$) the performances drop drastically (less than 40% ± 0.16 f1-score). On the contrary, for smaller subsets ($|S_{y}|\leq 5$), trained models obtain high recognition performances (more than 80% ± 0.05 f1-score). A deeper inspection of these configurations reveals that the location of selected data sources plays an important role; in particular, the latter subsets are mainly composed of hips data sources.

#### 6.3.1. Impact of Knowledge Derived Using Alternative Exploration Strategies

**Figure 23:**
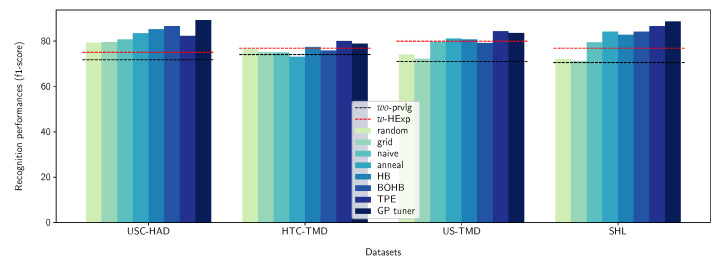
Impact of the architecture space exploration strategy used to derive privileged information incorporated into activity recognition models. Results obtained with the exploration strategies are listed in Section 6.2.1 are illustrated. Recognition performances, measured by the f1-score, of models trained with human expertise (*w*-HExp) and without any privileged information (*wo*-prvlg) are also illustrated.

In the previous experiment, we used the surrogate model based on the Gaussian process tuner in order to provide deployed activity recognition models with privileged information. The reason is that this surrogate model had the highest degree of agreement with domain experts. Depending on the exploration strategy used to select the experimental designs or hyperparameter instantiations to be evaluated, different regions of the architecture space will be favored, which will subsequently impact the knowledge that is derived from the surrogate model. That being said, even if the derived privileged information using these exploration strategies vary to larger extents, it will still capture the highly confident and informative data sources pertaining to each considered activity. For this, the effectiveness of privileged information derived using the exploration strategies listed in Section 6.2.1 is evaluated by using the same experimental setting used above for the Gaussian process exploration strategy. Figure 23 illustrates the obtained results for the four datasets which are considered here.

What we can observe is that in the case of the HTC-TMD and US-TMD datasets, the derived privileged information obtained using the tree-Parzen estimator (TPE) exploration strategy yields better activity recognition models than using Gaussian process, which, we recall, was the closest to human expertise. In that matter, it is worth observing that while the privileged information derived via exhaustive search strategies is the farthest from human expertise in terms of agreement, their incorporation into activity recognition models yields competitive results both on the HTC-TMD and USC-HAD datasets.

#### 6.3.2. Evaluation of the Robustness Using Dynamic Inductive Bias Selection

In this part, we evaluate the robustness of the learned models relative to the evolution of the sensing environments where these are deployed. We first evaluate the learned models in a continual setting (or 0-shot adaptation) and in a few-shot adaptation setting [119]. The scenarios featuring the evolution of the sensing environments correspond to the ablation of two or more sensors from the original set of sensors used in the SHL dataset.

##### Evaluation in a Continual Setting (0-Shot Adaptation)

**Figure 24:**
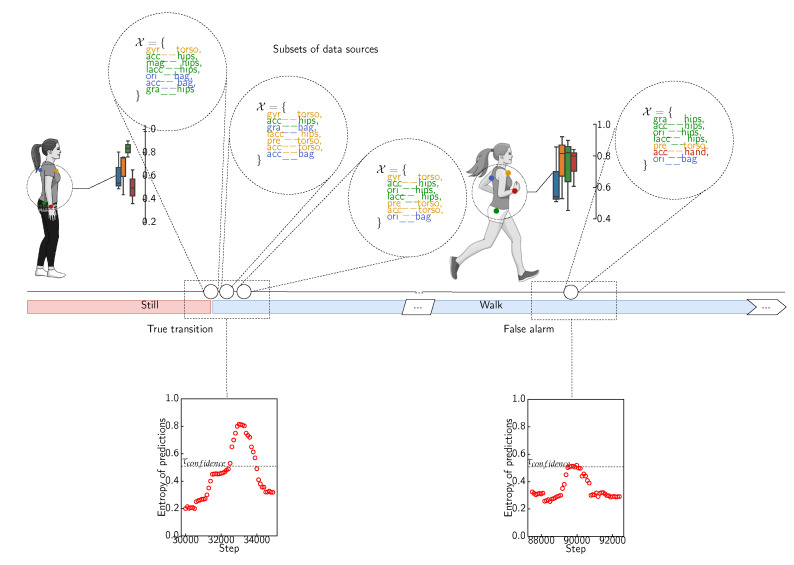
Timeline showing a real scenario of activity transition from *Still* (in red) to *Walk* (in blue). In addition to the true transition 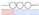, a false alarm 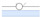 is triggered by the model during the walking activity. At the top of the timeline, the subsets of data sources are consecutively leveraged when the model detects a transition (true or false). At the bottom of the timeline, the two graphs show the evolution of the model’s predictions entropy monitored continuously against the confidence threshold ($\tau_{confidence}$). Figure adapted from [25].

The continual or zero-shot adaptation setting corresponds to the scenarios where no additional supervision is used to adapt or train the learning model in order to better cope with the new sensing configuration it is confronted with. We evaluate the behavior of the learned model from the perspective of robustness with regard to the phases encompassing transitions between activities. We assess the number of trials carried out by the model until the new activity, which we transitioned to, is recognized correctly. An additional parameter, referred to as the confidence threshold $\tau_{confidence}$, is considered in the analysis and defines the value of the model’s predictions entropy under which the predicted activity with the highest probability is considered to be correct ( the higher the entropy, the lower the model is confident about its predictions). Figure 24 illustrates one of the activity transition scenarios being evaluated.

##### Evaluation in a Few-Shot Adaptation Setting

**Figure 25:**
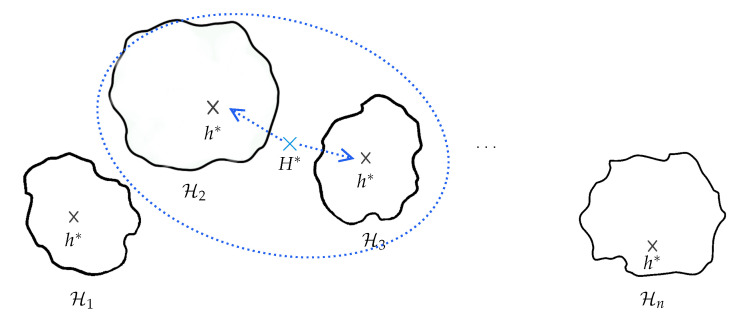
Illustration of the process of adaptation from the meta-hypothesis $H^{*}$ towards a more appropriate hypothesis $h^{*}$ (among those derived from the surrogate model) that matches the sensing configuration encountered by the deployed learning system.

Contrary to the previous setting, here we evaluate the model’s ability to adapt to the new sensing configurations with additional supervision consisting in fine-tuning the models using one (1-shot), five (5-shot), and ten (10-shot) additional learning example(s). Figure 25 illustrates the process of few-shots adaptation from a meta-hypothesis towards a more appropriate hypothesis that matches the sensing configuration encountered by the deployed activity recognition model.

Table 6 summarizes the obtained recognition performances by using the few-shot adaptation setting in various sensing configurations. In particular, the deployed activity recognition models built with help of the privileged information derived from the surrogate model are able to cope with extremely adverse sensing environments. We can observe that the 5-shot adaptation setting is able to cope with up to the ablation of nine sensors from the original sensor deployment.

## 7. Discussion

The dynamic inductive bias selection perspective that we propose to apply to human activity recognition could be framed into two levels: (1) making explicit the inductive biases related to the complete activity recognition chain (domain knowledge) in the form of surrogate models and (2) maintaining alternative or competing learning configurations (inductive biases) by allowing easy and rapid adaptation relative to new configurations. The discussion here is framed around the importance of domain knowledge and the three pillars of our proposed approach which can be stated as questions: (i) which knowledge to encode; (ii) how to encode it; and (iii) how do we incorporate it into deployed models? To make the analysis complete, throughout the discussion, we provide concrete and detailed examples of domain knowledge and the way these could be represented and incorporated into learning models.

### 7.1. Which Knowledge to Encode?

In the proposed instantiation of the framework, we investigated how the dynamics of body movements encoded in an explicit manner can be incorporated into activity recognition models in order to improve recognition performances. The incorporation of prior knowledge, particularly the topology of the on-body sensor deployments into activity recognition models, holds an important place in the literature. Long lines of research proposed, for example, to leverage 3D body skeleton-based representations exist [93,94,95,96,97], ontologies [120,121], etc. That being said, other aspects can be modeled and incorporated into activity recognition models and can affect virtually every step in the recognition chain from the measurement process to the topology of the sensor deployment as well as the transmission mechanisms used between the nodes of the sensor deployments.

**Figure 26:**
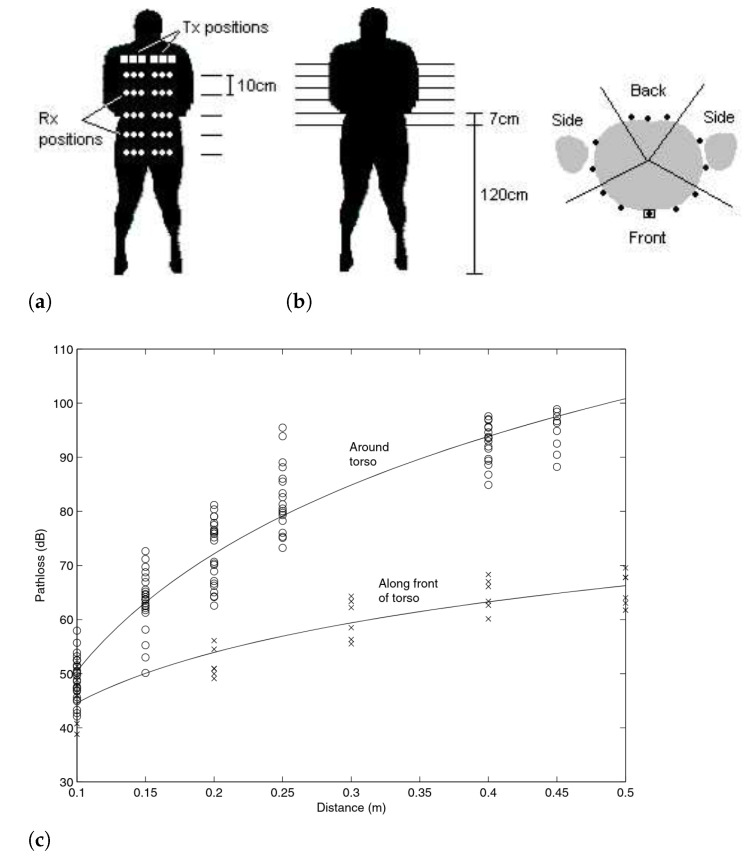
On-body placement of the sensing nodes (**a**) along the torso and (**b**) around the torso. (**c**) Measurement of the path loss (dB) as a function of the distance (m) between the sensing nodes around the torso (top line) and along the torso (bottom line). From [127].

Regarding these transmission mechanisms, the on-body placement of the sensing nodes has two visible and important interests: the first, as we saw, is related to global structure (or topology) that these nodes form and that we considered above to help us capture and leverage the dynamics of the body movements. The second is related to the physical layers of the radio-frequency (RF) communication components used to connect, in a wireless fashion, the various on-body sensors together and that are highly impacted by the placement of the sensing nodes. In particular, among these components, the radio channel forms the medium responsible for propagating raw data between the sensing nodes. This component is impacted by noise and interference which additionally evolves with time as a result, in the case of on-body sensor deployments, of the body movements and the environment (e.g., reflections of the radio waves on the walls) leading eventually to path loss and the impossibility to transmit data [122]. Various studies have been carried using different models of transceivers and showed a lack of communications among nodes depending on their on-body locations [123]. For examples, in [124,125], the authors experimented with 802.15.4-based CC2420 transceivers placed in different parts of the body including chest, ankle, and back of patients, etc. The results showed a lot of variations in terms of communication among the nodes. Figure 26 illustrates the impact of the transceivers’ on-body locations on the path loss. Furthermore, the authors in [126] studied the problem of path loss with respect to the underlying network topology, noticeably star vs. multi-hope mesh, where a reduction in the emitter-receiver distance could counteract this problem.

In addition to the impact of the on-body sensors placement on the path loss, the body movements as well as the surrounding environment have a big influence on signal propagation and subsequently on the packets transmissions. The authors in [127], for example, studied the influence of arm motions, while the authors in [128] considered the impact of various types of activities (still, walking, and running) on the path loss depending on the location of the transceivers. On this matter, Table 7 illustrates the shadowing standard deviation depending on the respective position of transmitters and receivers.

Similarly, the impact of the surrounding environment has been studied by the authors in [127] who studied signal propagation by taking into account factors related to the environment in which the user operates. These include, for example, the influence of ground reflections, which are considered more reliable in terms of being exploited during transmission, as well as reflections from surrounding environments on received signals.

As observed in Section 6.2.2 for the case of segmentation, the exhibition of hyperparameters can be extended to aspects other than the importance and interaction of data sources. Indeed, the long-term interest would be to make explicit the biases of all the stages of the activity recognition chain by going as far as the transfer functions which, as illustrated in the introduction of Section 4, constitute biases in their own.

In addition to the study of the domain knowledge, which is necessary for encoding, there is the important problem of the available resources to perform this operation. The sensing nodes have a limited autonomy, and storage and computational capacities, in particular, depend on this limit.

### 7.2. With What Resource Constraints?

Among the specificities and requirements of sensor deployments, which result in numerous constraints imposed on the operation of the applications they support, autonomy is probably the most important. The autonomy generates trade-offs involving the capacity of the nodes to sense and monitor (often in a continuous and near real-time fashion) the phenomenon being considered. Even by increasing battery capacities and optimizing components and processes, such as low-power hardware designs for the architectures, processors, and transceivers’ improvements [118,129], the problem is only shifted. Computing capacity constraints, backups, and direct consequences such as data sampling frequency, transmission frequency, and local processing must be taken into account in the general learning process and in sensor protocols [123]. For example, the authors in [130] presented an energy efficient, thermal-aware, and power-aware routing algorithm for on-body sensor deployments which considers the node’s temperature, energy level, and received power from adjacent nodes in the cost function calculation. Moreover, in [131], the authors investigated the selection of network interfaces, where the radio used to transmit is selected depending on the environment opportunities (bandwidth, link quality, and energy).

Another important problem is related to the heat generated by the sensors which sometimes modify the collected data by increasing the temperature of the body, for example. In [123], the authors investigated methods to restrict energy consumption and consequently to save the battery resources. In [132], the authors presented a temperature sensitive routing protocol in wireless body sensor networks for which temperature and heat production are fundamental. These routing protocols take the temperature of the node as a metric in the decision of the routing path. The purpose is to keep the temperature of the node below the safe level and to slow down the rate of temperature rise so that it does not harm the human body [132].

Although these trade-offs have a direct impact on the learning phase, they are often solely considered at the specific level where they arise. This makes it necessary to propagate these trade-offs, linked to material and IT aspects, to the level of learning processes. Some investigations [13,131,133] considered the direct link between energy/computational constraints with the performances of the activity recognition models. The authors in [13] investigated the trade-offs between classification accuracy and energy efficiency by comparing on-node and off-node schemes. An empirical energy model was presented and used to evaluate the energy efficiency of both systems, and a practical case study (monitoring the physical activities of office workers) was developed to evaluate the effect of classification accuracy. The results show that 40% energy saving can be obtained with a limited 13% reduction in classification accuracy. Similarly, with the goal of analyzing the trade-off between recognition accuracy and computational complexity, the authors in [133] investigated the impact of different sampling rates and other parameters on the performance of activity recognition models.

### 7.3. How to Encode (Represent) the Constraints and Knowledge?

The surrogate model is used as a proxy for the inductive biases of the activity recognition pipeline and particularly the data acquisition step. The proposed instantiation of the framework is based on neural architectures and the exploration of the space induced by the hyperparameters associated with these architectures. Making explicit these biases via hyperparameters is motivated by several aspects, the most important being their capacity to play the role of inductive biases far more than the parameters of a model. Indeed, the biases of the architecture (e.g., CNN for vision, LSTM for time-dependent sequences, etc.) are decisive for the tasks for which they were originally designed. More importantly, several empirical results are backing the fact that the hyperparameters are playing a more important role in the final recognition performances than the models’ parameters [92]. These results are also coherent with the direction of rapid adaptation and the use of few learning steps since the weights (parameters) of the models are less influential than the hyperparameters.

As we observed, the exploration of the architecture space is often based on an acquisition function (responsible of choosing the next configuration to explore); the entire issue is to design good acquisition functions that both have a good compromise between exploration and exploitation (which gives a fairly meaningful picture of the space of architectures) and at the same time reflect the targeted domain knowledge (the exploration must have the information that, for example, the targeted aspect is the bias related to segmentation, data sources, preprocessing, etc.). Concerning this point, the multi-modal architecture presented in Section 4.4.2 proceeds in this direction (the hyperparameters have been designed to reflect the impact of the data sources and their interactions).

### 7.4. How to Incorporate Knowledge into Deployed Models?

The method of incorporating knowledge, in a principled fashion, into deployed models remains also an open question as it is the case for the other aspects that we are investigating. Indeed, in the approach we proposed, it was not the best architecture that we have been interested in (and which would have been deployed directly without going through models of lower capacity) but rather the overall behavior of the architectures explored. This behavior was then incorporated into models that were more restricted in terms of capacity and, therefore, easy to train and adapt. That said, other methods of achieving this, by leveraging on existing techniques, could be implemented. In what follows, we present some avenues that can be investigated in this sense.

Regularization techniques where additional terms are plugged into the objective functions being optimized during model training have been investigated in the literature [134,135,136]. The principle is to train the model in a regular manner, i.e., minimize the objective function on learning examples, and additionally constrain the model to stay in some bounds defined by additional knowledge such as certain conditions, physical equation or laws, or first-order logic formulas. These techniques introduce new challenges for enforcing the simultaneous satisfaction of the terms of the objective function, i.e., the main term based on the learning examples and the additional regularization term, during the optimization process.

Attention mechanisms are additional computational levels that help neural networks to concentrate on the more important parts of the inputs in order to make predictions. These are widely used in natural language processing [137] and also in human activity recognition where, for example, the authors in [138] leveraged both temporal and sensor attention layers in order to help the recognition model to focus on more informative time steps of the inputs as well as on more important sensing modalities. These insights are learned simultaneously by the neural networks. These additional computational levels act on the structure of the neural networks by privileging the circuits that are attached to the data sources that are found to be the most informative with regard to the task of interest.

Sparsifying neural networks via pruning is also a method for incorporating accumulated knowledge. In [139], for example, the authors exploited sensitivity between inputs and outputs in order to eliminate model’s weights, which are not responsive enough to the input-output pairs stimulus during training. The pruning mechanism is widely used in neural networks training as a method of preventing the model from privileging a restricted number of its circuits, which could be more responsive to the input–output stimulus, and encouraging it to pursue diverse and alternative circuits. This again is a method of acting on the structure of the neural networks.

Neuromodulation in neural networks is concerned with the techniques that allow the structure of the neural networks to adapt according to certain high-level knowledge. For example, in [140], the authors proposed a neural architecture composed of two neural networks: a main network which processes ordinary data such as sensor data and a neuromodulatory network that is in charge of processing contextual data and feedback from the environment. Again, the idea here is that the neuromodulatory network, depending on the contextual data it processes, acts on the structure of the main neural networks in a manner that makes it more adapted to the environment it is confronted with.

## 8. Conclusions

We have shown in this paper that the study of data in the Internet of Things deployment should absolutely not be limited to the data generated by the sensors themselves at the risk of losing a significant amount of information resulting from the various biases and transformations that the data undergoes before arriving in the places where it is stored and processed. Therefore, it is necessary to consider all the transformations and distortions (biases) that the data undergoes and the context in which data collection takes place in order to build a representative learning or recognition framework. Moreover, other constraints related to energy consumption, waste heat generation, sensor topology, possible failures, and weak local resources add further biases and limit the types of solutions to consider. We have listed several constraints raised in sensor-rich environments, particularly with respect to on-body sensor deployments for activity recognition. We proposed a meta-modeling approach in which these constraints are specified as hyperparameters that can control the structure of the learning models. By using these hyperparameters, it was possible to optimize and reason about the various constraints that arise in these deployments, as well as incorporating prior knowledge into the learning processes. In particular, the exploration of the hyperparameter’s space and the analysis step conducted using the uncertainty quantification framework (Section 6.2) allowed drawing some links between environmental constraints and the structure of the learning models. These links are leveraged during model deployment to cope with noticeably the evolution of the sensing configurations. Extensive experiments on a use-case pertaining to the SHL dataset illustrated the advantages of the proposed approach. These results make the case for the proposed meta-modeling approach and show the robustness gains achieved when the deployed models are confronted with evolution of the initial sensing configurations (ablation of an increasing number of sensors from the initial deployment). In particular, incorporation of the derived knowledge about the sensors deployment allows easy adaptation using little or no supervision at all. This work opens-up perspectives for developing more robust and reliable learning systems in the Internet of things.

## Figures and Tables

**Table 1:** Hyperparameters of the architectural components and their associated ranges used for the construction of the surrogate model. From [109].

Hyperparameter	Low	High	Prior
Learning rate (lr)	0.001	0.1	log
Kernel size 1st ($ks_{1}$)	9	15	-
Kernel size 2nd ($ks_{2}$)	9	15	-
Kernel size 3rd ($ks_{3}$)	9	12	-
Number of filters ($n_{f}$)	16	28	-
Stride (*s*)	0.5	0.6	log
Number of units dense layer ($n_{u}$)	64	2048	-
Number of hidden units 1 ($n_{hu1}$)	64	384	-
Number of hidden units 2 ($n_{hu2}$)	64	384	-
Inputs dropout probability ($p_{in}$)	0.5	1	log
Outputs dropout probability ($p_{ou}$)	0.5	1	log
States dropout probability ($p_{st}$)	0.5	1	log

**Table 2:** Summary of the results obtained with the fANOVA analysis showing the importance of the individual importance of the hyperparameters associated with the architectures defined in Section 6.2.1. Architectures with recurrent output layers are referred to as *Hybrid* while those with dense output layers are referred to as *Convolutional*. From [109].

Hyperparameter	Individual Importance	
	Convolutional	Hybrid
Learning rate ($l_{r}$)	0.10423	0.19815
Kernel size 1st ($ks_{1}$)	0.01410	0.00874
Kernel size 2nd ($ks_{2}$)	0.00916	0.023105
Kernel size 3rd ($ks_{3}$)	0.04373	0.01788
Number of filters ($n_{f}$)	0.02810	0.01845
Stride (*s*)	0.08092	0.06236
Number of units dense layer ($n_{u}$)	0.16748	-
Number of hidden units 1 ($n_{hu1}$)	-	0.06324
Number of hidden units 2 ($n_{hu2}$)	-	0.02478
Inputs dropout probability ($p_{in}$)	-	0.04047
Outputs dropout probability ($p_{ou}$)	-	0.01056
States dropout probability ($p_{st}$)	-	0.01991

**Table 3:** Level of agreement between privileged information (or knowledge) derived from the surrogate model and human expertise by using different space exploration strategies. The level of agreement with the human expertise-based model (HExp) is measured using Cohen’s kappa coefficient [117]. The partial recognition performances $\nu_{k}$ obtained while exploring the space are averaged and illustrated. From [23].

Exploration Strategy	Agreement	$\nu_{k}$ on Average
Exhaustive search		
Random Search	0.156 ± 0.04	67.12%
Grid Search	**0.251 ± 0.05**	66.78%
Heuristic search		
Naïve evolution	0.347 ± 0.12	73.35%
Anneal	**0.481 ± 0.05**	75.47%
Hyperband	0.395 ± 0.08	74.2%
Sequential Model-Based		
BOHB	0.734 ± 0.03	84.25%
TPE	0.645 ± 0.1	83.87%
GP Tuner	**0.865 ± 0.02**	84.95%

**Table 4:** Details of the datasets used to evaluate the impact of incorporating the derived surrogate model of the data acquisition step into activity recognition models. Illustrated details include the availability of multiple modalities in multiple locations simultaneously. Motion-based modalities, which are referred to with the following abbreviations: acc (accelerometer), gyr (gyroscope), mag (magnetometer), lac (linear accelerometer) gra (gravity) ori (orientation), and pre (pressure). In addition, GPS refers to global positioning system, GSR to galvanic skin response, and ECG to electrocardiogram.

Dataset/Study	Multi-Modality	Multi-Location	Modality	Location	Activity
USC-HAD [20]	✔	✗	acc, gyr, mag, GPS, WiFi, ECG, GSR, etc.	hip	walking forward, walking left, walking right, walking upstairs, walking downstairs, running forward, jumping, sitting, standing, sleeping, elevator up, and elevator down
HTC-TMD [21]	✔	✗	acc, gyr, mag, GPS, WiFi	-	Still, Walk, Run, Bike, (riding) Motorcycle, Car, Bus, Metro, Train, and high speed rail (HSR)
US-TMD [22]	✔	✗	acc, gyr, sound	-	Walking, Car, Still, Train, and Bus
w-HAR [118]	✔	✔	IMU (accelerometer and gyroscope) and stretch sensor	ankle, knee	jump, lie down, sit, stand, stairs down, stairs up, and walk
SHL [18]	✔	✔	acc, gyr, mag, lacc, ori, gra, etc.	hand, hips, bag, torso	Still, Walk, Run, Bike, Car, Bus, Train, and Subway

**Table 5:** Comparison of different privileged settings in terms of recognition performances on four different datasets USC-HAD, HTC-TMD, US-TMD, and SHL. Privileged information are derived from the surrogate model constructed by using the data featured in the SHL dataset. Scores of column *w*-prvlg correspond to top-performing models selected while varying the data source importance threshold $\tau_{imp}$. Recognition performance means and variances are reported on a total of 10 experiments repetitions.

Dataset	Recognition Performances		
	*wo*-Prvlg	*w*-HExp	*w*-Prvlg
USC-HAD	72.1% ± 0.009	75.38% ± 0.33	89.33% ± 0.14
HTC-TMD	74.4% ± 0.03	77.16% ± 0.5	78.9% ± 0.067
US-TMD	71.32% ± 0.2	80.28% ± 0.008	83.64% ± 0.04
SHL	70.86% ± 0.12	77.18% ± 0.044	88.7% ± 0.6

**Table 6:** Few-shot recognition performances on various sensing configurations featuring the ablation of 2, 5, 9, and 12 sensors from the original sensor deployment of the SHL dataset. For reference, the baseline evaluated on the sensor ablation configurations is also shown.

Configuration	Baseline	Surrogate-Informed		
		1-Shot	5-Shot	10-Shot
2-sensor ablation	69.56% ± 0.074	88.09% ± 0.01	88.51% ± 0.081	89.55% ± 0.09
5-sensor ablation	62.17.4% ± 0.08	86.12% ± 0.047	87.92% ± 0.07	87.88% ± 0.16
9-sensor ablation	51.22% ± 0.026	81.27% ± 0.204	85.23% ± 0.14	86.08% ± 0.097
12-sensor ablation	41.02 % ± 0.84	75.74 % ± 0.51	82.17% ± 0.37	82.32% ± 0.64

**Table 7:** Measurement of the shadowing standard deviation (dB) in anechoic chamber and indoor. Various on-body placement configuration assessed. From [128].

TX on Hip/RX on	Chest	Thigh	R Wrist	R Foot
Anechoic chamber				
Still	0.61	0.41	0.95	0.41
Walking	2.15	5.27	4.31	4.97
Running	2.39	2.47	3.49	2.69
Indoor				
Still	0.60	0.24	0.26	0.24
Walking	1.52	3.27	2.66	2.57
Running	2.00	1.98	2.37	1.80
TX on L Ear/RX on	R Ear	Hip	R Wrist	R Foot
Anechoic chamber				
Still	0.23	0.45	1.15	0.56
Walking	0.54	2.07	3.35	4.21
Running	0.52	2.82	2.98	1.57
Indoor				
Still	0.21	0.28	0.31	0.30
Walking	0.90	2.20	1.80	2.02
Running	0.70	2.19	1.88	1.24

## Data Availability

Not applicable.

## References

[1] Abdallah Z.S., Gaber M.M., Srinivasan B., Krishnaswamy S. (2018). Activity recognition with evolving data streams: A review. ACM Comput. Surv. (CSUR).

[2] Aliverti A. (2017). Wearable technology: Role in respiratory health and disease. Breathe.

[3] Lussier M., Aboujaoudé A., Couture M., Moreau M., Laliberté C., Giroux S., Pigot H., Gaboury S., Bouchard K., Belchior P. (2020). Using ambient assisted living to monitor older adults with alzheimer disease: Single-case study to validate the monitoring report. JMIR Med. Inform..

[4] Dang L.M., Min K., Wang H., Piran M.J., Lee C.H., Moon H. (2020). Sensor-based and vision-based human activity recognition: A comprehensive survey. Pattern Recognit..

[5] Andreu-Perez J., Leff D.R., Ip H.M., Yang G.Z. (2015). From wearable sensors to smart implants—toward pervasive and personalized healthcare. IEEE Trans. Biomed. Eng..

[6] Spiegel B.M., Kaneshiro M., Russell M.M., Lin A., Patel A., Tashjian V.C., Zegarski V., Singh D., Cohen S.E., Reid M.W. (2014). Validation of an acoustic gastrointestinal surveillance biosensor for postoperative ileus. J. Gastrointest. Surg..

[7] Singh A., Rehman S.U., Yongchareon S., Chong P.H.J. (2020). Sensor technologies for fall detection systems: A review. IEEE Sens. J..

[8] Marcello F., Pilloni V. (2020). Smart Building Energy and Comfort Management Based on Sensor Activity Recognition and Prediction. Sensors.

[9] Dong X.L., Rekatsinas T. Data integration and machine learning: A natural synergy. Proceedings of the 2018 International Conference on Management of Data.

[10] Attal F., Mohammed S., Dedabrishvili M., Chamroukhi F., Oukhellou L., Amirat Y. (2015). Physical human activity recognition using wearable sensors. Sensors.

[11] Mainetti L., Patrono L., Vilei A. Evolution of wireless sensor networks towards the internet of things: A survey. Proceedings of the 19th International Conference on Software, Telecommunications and Computer Networks, SoftCOM 2011.

[12] Ida N. (2020). Sensors, Actuators, and Their Interfaces: A Multidisciplinary Introduction.

[13] Wang N., Merrett G.V., Maunder R.G., Rogers A. Energy and accuracy trade-offs in accelerometry-based activity recognition. Proceedings of the 2013 22nd International Conference on Computer Communication and Networks (ICCCN).

[14] Krishnamachari B. (2005). Networking Wireless Sensors.

[15] Abidi B., Jilbab A., Mohamed E.H. (2020). Wireless body area networks: A comprehensive survey. J. Med. Eng. Technol..

[16] Bulling A., Blanke U., Schiele B. (2014). A tutorial on human activity recognition using body-worn inertial sensors. ACM Comput. Surv. (CSUR).

[17] Mitchell T.M. (1980). The Need for Biases in Learning Generalizations.

[18] Gjoreski H., Ciliberto M., Wang L., Ordonez Morales F.J., Mekki S., Valentin S., Roggen D. (2018). The University of Sussex-Huawei locomotion and transportation dataset for multimodal analytics with mobile devices. IEEE Access.

[19] Zheng Y., Xie X., Ma W.Y. (2010). Geolife: A collaborative social networking service among user, location and trajectory. IEEE Data Eng. Bull..

[20] Zhang M., Sawchuk A.A. USC-HAD: A daily activity dataset for ubiquitous activity recognition using wearable sensors. Proceedings of the 2012 ACM Conference on Ubiquitous Computing.

[21] Yu M.C., Yu T., Wang S.C., Lin C.J., Chang E.Y. (2014). Big data small footprint: The design of a low-power classifier for detecting transportation modes. Proc. VLDB Endow..

[22] Carpineti C., Lomonaco V., Bedogni L., Di Felice M., Bononi L. Custom Dual Transportation Mode Detection by Smartphone Devices Exploiting Sensor Diversity. Proceedings of the 2018 IEEE International Conference on Pervasive Computing and Communications Workshops (PerCom Workshops).

[23] Hamidi M., Osmani A. Data Generation Process Modeling for Activity Recognition. Proceedings of the European Conference on Machine Learning and Principles and Practice of Knowledge Discovery in Databases.

[24] Osmani A., Hamidi M. Hybrid and convolutional neural networks for locomotion recognition. Proceedings of the 2018 ACM UbiComp/ISWC 2018 Adjunct.

[25] Hamidi M., Osmani A. (2020). Domain Models for Data Sources Integration in HAR. Neurocomputing.

[26] Aggarwal J.K., Ryoo M.S. (2011). Human activity analysis: A review. ACM Comput. Surv. (CSUR).

[27] Jiang W., Miao C., Ma F., Yao S., Wang Y., Yuan Y., Xue H., Song C., Ma X., Koutsonikolas D. Towards environment independent device free human activity recognition. Proceedings of the 24th Annual International Conference on Mobile Computing and Networking.

[28] Beddiar D.R., Nini B., Sabokrou M., Hadid A. (2020). Vision-based human activity recognition: A survey. Multimed. Tools Appl..

[29] Demrozi F., Pravadelli G., Bihorac A., Rashidi P. (2020). Human activity recognition using inertial, physiological and environmental sensors: A comprehensive survey. IEEE Access.

[30] Li F., Shirahama K., Nisar M.A., Köping L., Grzegorzek M. (2018). Comparison of feature learning methods for human activity recognition using wearable sensors. Sensors.

[31] Aghajan H., Cavallaro A. (2009). Multi-Camera Networks: Principles and Applications.

[32] Wu C., Khalili A.H., Aghajan H. Multiview activity recognition in smart homes with spatio-temporal features. Proceedings of the Fourth ACM/IEEE International Conference on Distributed Smart Cameras.

[33] Hussain T., Muhammad K., Ullah A., Del Ser J., Gandomi A.H., Sajjad M., Baik S.W., de Albuquerque V.H.C. (2020). Multiview Summarization and Activity Recognition Meet Edge Computing in IoT Environments. IEEE Internet Things J..

[34] Nguyen P., Ferry N., Erdogan G., Song H., Lavirotte S., Tigli J.Y., Solberg A. Advances in deployment and orchestration approaches for IoT-a systematic review. Proceedings of the 2019 IEEE International Congress on Internet of Things (ICIOT).

[35] Wang W., Liu A.X., Shahzad M., Ling K., Lu S. (2017). Device-free human activity recognition using commercial WiFi devices. IEEE J. Sel. Areas Commun..

[36] Wang Z., Jiang K., Hou Y., Dou W., Zhang C., Huang Z., Guo Y. (2019). A survey on human behavior recognition using channel state information. IEEE Access.

[37] Chowdhury T.Z. (2018). Using Wi-Fi Channel State Information (CSI) for Human Activity Recognition and Fall Detection. Ph.D. Thesis.

[38] Qian K., Wu C., Yang Z., Liu Y., He F., Xing T. (2018). Enabling contactless detection of moving humans with dynamic speeds using CSI. ACM Trans. Embed. Comput. Syst. (TECS).

[39] Zhou Q., Xing J., Li J., Yang Q. A device-free number gesture recognition approach based on deep learning. Proceedings of the 2016 12th International Conference on Computational Intelligence and Security (CIS).

[40] Arshad S., Feng C., Liu Y., Hu Y., Yu R., Zhou S., Li H. Wi-chase: A WiFi based human activity recognition system for sensorless environments. Proceedings of the 2017 IEEE 18th International Symposium on A World of Wireless, Mobile and Multimedia Networks (WoWMoM).

[41] Cohen L. (1995). Uncertainty principles of the short-time Fourier transform. Advanced Signal Processing Algorithms.

[42] Zhang F., Niu K., Xiong J., Jin B., Gu T., Jiang Y., Zhang D. (2019). Towards a diffraction-based sensing approach on human activity recognition. Proc. ACM Interact. Mob. Wearable Ubiquitous Technol..

[43] He W., Wu K., Zou Y., Ming Z. WiG: WiFi-based gesture recognition system. Proceedings of the 2015 24th International Conference on Computer Communication and Networks (ICCCN).

[44] Yala N., Fergani B., Fleury A. Feature extraction for human activity recognition on streaming data. Proceedings of the 2015 International Symposium on Innovations in Intelligent SysTems and Applications (INISTA).

[45] Shoaib M., Bosch S., Incel O.D., Scholten H., Havinga P.J. (2016). Complex human activity recognition using smartphone and wrist-worn motion sensors. Sensors.

[46] Banos O., Galvez J.M., Damas M., Pomares H., Rojas I. (2014). Window size impact in human activity recognition. Sensors.

[47] Osmani A., Hamidi M., Chibani A. Platform for assessment and monitoring of infant comfort. Proceedings of the 2017 AAAI Fall Symposium Series.

[48] Hammerla N.Y., Plötz T. (2015). Let’s (not) stick together: Pairwise similarity biases cross-validation in activity recognition. Proceedings of the 2015 ACM International Joint Conference on Pervasive and Ubiquitous Computing.

[49] Millecamps A., Lowry K.A., Brach J.S., Perera S., Redfern M.S., Sejdić E. (2015). Understanding the effects of pre-processing on extracted signal features from gait accelerometry signals. Comput. Biol. Med..

[50] Harris F.J. (1978). On the use of windows for harmonic analysis with the discrete Fourier transform. Proc. IEEE.

[51] Boris M. ADC Performance Survey 1997–2017. http://web.stanford.edu/~murmann/adcsurvey.html.

[52] Danial L., Wainstein N., Kraus S., Kvatinsky S. (2018). Breaking through the speed-power-accuracy tradeoff in ADCs using a memristive neuromorphic architecture. IEEE Trans. Emerg. Top. Comput. Intell..

[53] Ma Y., Ghasemzadeh H. An asynchronous multi-view learning approach for activity recognition using wearables. Proceedings of the 2016 38th Annual International Conference of the IEEE Engineering in Medicine and Biology Society (EMBC).

[54] Elhabyan R., Shi W., St-Hilaire M. (2019). Coverage protocols for wireless sensor networks: Review and future directions. J. Commun. Netw..

[55] Chung S., Lim J., Noh K.J., Kim G.G., Jeong H.T. Sensor positioning and data acquisition for activity recognition using deep learning. Proceedings of the 2018 International Conference on Information and Communication Technology Convergence (ICTC).

[56] Gjoreski H., Gams M. Accelerometer data preparation for activity recognition. Proceedings of the International Multiconference Information Society.

[57] Karantonis D.M., Narayanan M.R., Mathie M., Lovell N.H., Celler B.G. (2006). Implementation of a real-time human movement classifier using a triaxial accelerometer for ambulatory monitoring. IEEE Trans. Inf. Technol. Biomed..

[58] Mathie M.J., Celler B.G., Lovell N.H., Coster A.C. (2004). Classification of basic daily movements using a triaxial accelerometer. Med. Biol. Eng. Comput..

[59] Parkka J., Ermes M., Korpipaa P., Mantyjarvi J., Peltola J., Korhonen I. (2006). Activity classification using realistic data from wearable sensors. IEEE Trans. Inf. Technol. Biomed..

[60] Yang J.Y., Wang J.S., Chen Y.P. (2008). Using acceleration measurements for activity recognition: An effective learning algorithm for constructing neural classifiers. Pattern Recognit. Lett..

[61] Chung S., Lim J., Noh K.J., Kim G., Jeong H. (2019). Sensor data acquisition and multimodal sensor fusion for human activity recognition using deep learning. Sensors.

[62] Lawal I.A., Bano S. Deep human activity recognition using wearable sensors. Proceedings of the 12th ACM International Conference on PErvasive Technologies Related to Assistive Environments.

[63] Sztyler T., Stuckenschmidt H. On-body localization of wearable devices: An investigation of position-aware activity recognition. Proceedings of the 2016 IEEE International Conference on Pervasive Computing and Communications (PerCom).

[64] Bao L., Intille S.S. Activity recognition from user-annotated acceleration data. Proceedings of the International Conference on Pervasive Computing.

[65] Gao L., Bourke A., Nelson J. (2014). Evaluation of accelerometer based multi-sensor versus single-sensor activity recognition systems. Med. Eng. Phys..

[66] Han F., Liu X., Mohamed I.I., Ghazali K.H., Zhao Y. A Survey on Deployment and Coverage Strategies in Three-Dimensional Wireless Sensor Networks. Proceedings of the 2019 8th International Conference on Software and Computer Applications.

[67] Förster K., Brem P., Roggen D., Tröster G. Evolving discriminative features robust to sensor displacement for activity recognition in body area sensor networks. Proceedings of the 2009 International Conference on Intelligent Sensors, Sensor Networks and Information Processing (ISSNIP).

[68] Kunze K., Lukowicz P. Dealing with sensor displacement in motion-based onbody activity recognition systems. Proceedings of the 10th International Conference on Ubiquitous Computing.

[69] Banos O., Toth M.A., Damas M., Pomares H., Rojas I. (2014). Dealing with the effects of sensor displacement in wearable activity recognition. Sensors.

[70] Shi J., Zuo D., Zhang Z., Luo D. (2020). Sensor-based activity recognition independent of device placement and orientation. Trans. Emerg. Telecommun. Technol..

[71] Barshan B., Yurtman A. (2020). Classifying Daily and Sports Activities Invariantly to the Positioning of Wearable Motion Sensor Units. IEEE Internet Things J..

[72] Stisen A., Blunck H., Bhattacharya S., Prentow T.S., Kjærgaard M.B., Dey A., Sonne T., Jensen M.M. Smart devices are different: Assessing and mitigatingmobile sensing heterogeneities for activity recognition. Proceedings of the 13th ACM Conference on Embedded Networked Sensor Systems.

[73] Baldominos A., Saez Y., Isasi P. (2018). Evolutionary design of convolutional neural networks for human activity recognition in sensor-rich environments. Sensors.

[74] Wang A., Chen G., Yang J., Zhao S., Chang C.Y. (2016). A comparative study on human activity recognition using inertial sensors in a smartphone. IEEE Sens. J..

[75] Stiefmeier T., Ogris G., Junker H., Lukowicz P., Troster G. Combining motion sensors and ultrasonic hands tracking for continuous activity recognition in a maintenance scenario. Proceedings of the 2006 10th IEEE International Symposium on Wearable Computers.

[76] Pentney S.W.W., Popescu A.M., Choudhury T., Philipose M. Common sense based joint training of human activity recognizers. Proceedings of the 20th International Joint Conference on Artificial Intelligence.

[77] Yao S., Hu S., Zhao Y., Zhang A., Abdelzaher T. Deepsense: A unified deep learning framework for time-series mobile sensing data processing. Proceedings of the International Conference on World Wide Web.

[78] Ha S., Choi S. Convolutional neural networks for human activity recognition using multiple accelerometer and gyroscope sensors. Proceedings of the 2016 International Joint Conference on Neural Networks (IJCNN).

[79] Xing T., Sandha S.S., Balaji B., Chakraborty S., Srivastava M. Enabling edge devices that learn from each other: Cross modal training for activity recognition. Proceedings of the 1st International Workshop on Edge Systems, Analytics and Networking.

[80] Chen K., Zhang D., Yao L., Guo B., Yu Z., Liu Y. (2021). Deep Learning for Sensor-based Human Activity Recognition: Overview, Challenges, and Opportunities. ACM Comput. Surv. (CSUR).

[81] Trusov A.A., Zotov S.A., Simon B.R., Shkel A.M. Silicon accelerometer with differential frequency modulation and continuous self-calibration. Proceedings of the 2013 IEEE 26th International Conference on Micro Electro Mechanical Systems (MEMS).

[82] De Campos Porath M., Dolci R. (2015). Uncertainty of angular displacement measurement with a MEMS gyroscope integrated in a smartphone. J. Phys. Conf. Ser..

[83] Vapnik V.N. (1995). The Nature of Statistical Learning Theory.

[84] Baxter J. (2000). A model of inductive bias learning. J. Artif. Intell. Res..

[85] Vapnik V.N., Chervonenkis A.Y. (1982). Necessary and sufficient conditions for the uniform convergence of means to their expectations. Theory Probab. Its Appl..

[86] Valiant L.G. (1984). A theory of the learnable. Commun. ACM.

[87] Blumer A., Ehrenfeucht A., Haussler D., Warmuth M.K. (1989). Learnability and the Vapnik-Chervonenkis dimension. J. ACM (JACM).

[88] Utgoff P.E. (1986). Machine Learning of Inductive Bias.

[89] Garipov T., Izmailov P., Podoprikhin D., Vetrov D., Wilson A.G. Loss surfaces, mode connectivity, and fast ensembling of DNNs. Proceedings of the 32nd International Conference on Neural Information Processing Systems.

[90] Sudret B., Marelli S., Wiart J. Surrogate models for uncertainty quantification: An overview. Proceedings of the 2017 11th European Conference on Antennas and Propagation (EUCAP).

[91] Sudret B. (2007). Uncertainty Propagation and Sensitivity Analysis in Mechanical Models–Contributions to Structural Reliability and Stochastic Spectral Methods. Ph.D. Thesis.

[92] Gaier A., Ha D. Weight agnostic neural networks. Proceedings of the Advances in Neural Information Processing Systems.

[93] Vatavu R.D., Pentiuc S.G. (2012). Multi-level representation of gesture as command for human computer interaction. Comput. Inform..

[94] Kovalenko M., Antoshchuk S., Sieck J. Real-time hand tracking and gesture recognition using semantic-probabilistic network. Proceedings of the 2014 UKSim-AMSS 16th International Conference on Computer Modelling and Simulation.

[95] Papadopoulos G.T., Axenopoulos A., Daras P. Real-time skeleton-tracking-based human action recognition using kinect data. Proceedings of the International Conference on Multimedia Modeling.

[96] Parisi G.I., Tani J., Weber C., Wermter S. (2017). Emergence of multimodal action representations from neural network self-organization. Cogn. Syst. Res..

[97] Dhiman C., Vishwakarma D.K., Aggarwal P. (2019). Skeleton based Activity Recognition by Fusing Part-wise Spatio-temporal and Attention Driven Residues. arXiv.

[98] Kleijnen J.P. (2009). Kriging metamodeling in simulation: A review. Eur. J. Oper. Res..

[99] Elsken T., Metzen J.H., Hutter F. (2019). Neural Architecture Search: A Survey. J. Mach. Learn. Res..

[100] Hoeffding W. (1948). A non-parametric test of independence. Ann. Math. Stat..

[101] Vapnik V., Izmailov R. (2015). Learning using privileged information: Similarity control and knowledge transfer. J. Mach. Learn. Res..

[102] Hinton G., Vinyals O., Dean J. (2015). Distilling the knowledge in a neural network. arXiv.

[103] Lopez-Paz D., Bottou L., Schölkopf B., Vapnik V. (2015). Unifying distillation and privileged information. arXiv.

[104] Ordóñez F.J., Roggen D. (2016). Deep convolutional and lstm recurrent neural networks for multimodal wearable activity recognition. Sensors.

[105] Radu V., Tong C., Bhattacharya S., Lane N.D., Mascolo C., Marina M.K., Kawsar F. (2018). Multimodal deep learning for activity and context recognition. Proc. ACM Interact. Mob. Wearable Ubiquitous Technol..

[106] Bevilacqua A., MacDonald K., Rangarej A., Widjaya V., Caulfield B., Kechadi T. Human Activity Recognition with Convolutional Neural Networks. Proceedings of the Joint European Conference on Machine Learning and Knowledge Discovery in Databases.

[107] Pham H., Guan M., Zoph B., Le Q., Dean J. Efficient Neural Architecture Search via Parameters Sharing. Proceedings of the International Conference on Machine Learning.

[108] Liu H., Simonyan K., Yang Y. DARTS: Differentiable Architecture Search. Proceedings of the International Conference on Learning Representations.

[109] Osmani A., Hamidi M. (2019). Bayesian Optimization of Neural Architectures for Human Activity Recognition. Human Activity Sensing.

[110] Bergstra J., Bengio Y. (2012). Random search for hyper-parameter optimization. J. Mach. Learn. Res..

[111] Real E., Moore S., Selle A., Saxena S., Suematsu Y.L., Tan J., Le Q.V., Kurakin A. Large-scale evolution of image classifiers. Proceedings of the 34th International Conference on Machine Learning.

[112] Bergstra J.S., Bardenet R., Bengio Y., Kégl B. Algorithms for hyper-parameter optimization. Proceedings of the Advances in Neural Information Processing Systems.

[113] Li L., Jamieson K., DeSalvo G., Rostamizadeh A., Talwalkar A. (2017). Hyperband: A novel bandit-based approach to hyperparameter optimization. J. Mach. Learn. Res..

[114] Falkner S., Klein A., Hutter F. (2018). BOHB: Robust and efficient hyperparameter optimization at scale. arXiv.

[115] Hoos H., Leyton-Brown K. An efficient approach for assessing hyperparameter importance. Proceedings of the International Conference on Machine Learning.

[116] Muehlenstaedt T., Roustant O., Carraro L., Kuhnt S. (2012). Data-driven Kriging models based on FANOVA-decomposition. Stat. Comput..

[117] Cohen J. (1960). A coefficient of agreement for nominal scales. Educ. Psychol. Meas..

[118] Bhat G., Tran N., Shill H., Ogras U.Y. (2020). w-HAR: An activity recognition dataset and framework using low-power wearable devices. Sensors.

[119] Finn C., Abbeel P., Levine S. Model-Agnostic Meta-Learning for Fast Adaptation of Deep Networks. Proceedings of the International Conference on Machine Learning.

[120] Ousmer M., Vanderdonckt J., Buraga S. An ontology for reasoning on body-based gestures. Proceedings of the ACM SIGCHI Symposium on Engineering Interactive Computing Systems.

[121] Rodríguez N.D., Wikström R., Lilius J., Cuéllar M.P., Flores M.D.C. Understanding movement and interaction: An ontology for Kinect-based 3D depth sensors. Proceedings of the Ubiquitous Computing and Ambient Intelligence. Context-Awareness and Context-Driven Interaction.

[122] Goldsmith A. (2005). Path Loss and Shadowing. Wireless Communications.

[123] Latré B., Braem B., Moerman I., Blondia C., Demeester P. (2011). A survey on wireless body area networks. Wirel. Netw..

[124] Ruzzelli A.G., Jurdak R., O’Hare G.M., Van Der Stok P. Energy-efficient multi-hop medical sensor networking. Proceedings of the 1st ACM SIGMOBILE International Workshop on Systems and Networking Support for Healthcare and Assisted Living Environments.

[125] Shah R.C., Yarvis M. Characteristics of on-body 802.15. 4 networks. Proceedings of the 2006 2nd IEEE Workshop on Wireless Mesh Networks.

[126] Gorce J.M., Goursaud C., Villemaud G., d’Errico R., Ouvry L. Opportunistic relaying protocols for human monitoring in BAN. Proceedings of the 2009 IEEE 20th International Symposium on Personal, Indoor and Mobile Radio Communications.

[127] Fort A., Desset C., Ryckaert J., De Doncker P., Van Biesen L., Wambacq P. Characterization of the ultra wideband body area propagation channel. Proceedings of the 2005 IEEE International Conference on Ultra-Wideband.

[128] D’Errico R., Ouvry L. Time-variant BAN channel characterization. Proceedings of the 2009 IEEE 20th International Symposium on Personal, Indoor and Mobile Radio Communications.

[129] Sudevalayam S., Kulkarni P. (2010). Energy harvesting sensor nodes: Survey and implications. IEEE Commun. Surv. Tutorials.

[130] Movassaghi S., Abolhasan M., Lipman J. Energy efficient thermal and power aware (ETPA) routing in body area networks. Proceedings of the 2012 IEEE 23rd International Symposium on Personal, Indoor and Mobile Radio Communications (PIMRC).

[131] Rault T., Bouabdallah A., Challal Y., Marin F. (2017). A survey of energy-efficient context recognition systems using wearable sensors for healthcare applications. Pervasive Mob. Comput..

[132] Oey C.H.W., Moh S. (2013). A survey on temperature-aware routing protocols in wireless body sensor networks. Sensors.

[133] Maurer U., Smailagic A., Siewiorek D.P., Deisher M. Activity recognition and monitoring using multiple sensors on different body positions. Proceedings of the International Workshop on Wearable and Implantable Body Sensor Networks (BSN’06).

[134] Stewart R., Ermon S. Label-free supervision of neural networks with physics and domain knowledge. Proceedings of the Thirty-First AAAI Conference on Artificial Intelligence.

[135] Nabian M.A., Meidani H. (2020). Physics-Driven Regularization of Deep Neural Networks for Enhanced Engineering Design and Analysis. J. Comput. Inf. Sci. Eng..

[136] Osmani A., Hamidi M., Bouhouche S. Augmented Experiment in Material Engineering Using Machine Learning. Proceedings of the Thirty-Fifth AAAI Conference on Artificial Intelligence, Virtual Event.

[137] Bahdanau D., Cho K., Bengio Y. (2014). Neural machine translation by jointly learning to align and translate. arXiv.

[138] Zeng M., Gao H., Yu T., Mengshoel O.J., Langseth H., Lane I., Liu X. Understanding and improving recurrent networks for human activity recognition by continuous attention. Proceedings of the 2018 ACM International Symposium on Wearable Computers.

[139] Tartaglione E., Lepsøy S., Fiandrotti A., Francini G. (2018). Learning sparse neural networks via sensitivity-driven regularization. Adv. Neural Inf. Process. Syst..

[140] Vecoven N., Ernst D., Wehenkel A., Drion G. (2020). Introducing neuromodulation in deep neural networks to learn adaptive behaviours. PLoS ONE.

